# *Bacteroides ovatus* accelerates metformin-induced vitamin B12 deficiency in type 2 diabetes patients by accumulating cobalamin

**DOI:** 10.1038/s41522-023-00419-y

**Published:** 2023-07-24

**Authors:** Manyun Chen, Yan Shu, Qing Li, Zhiqiang Kang, Tao Liu, Honghao Zhou, Weihua Huang, Wei Zhang

**Affiliations:** 1 Department of Clinical Pharmacology, Xiangya Hospital, Central South University, Changsha, P. R. China; 2 Institute of Clinical Pharmacology, Central South University, Hunan Key Laboratory of Pharmacogenetics, Changsha, P. R. China; 3 Engineering Research Center of Applied Technology of Pharmacogenomics, Ministry of Education, Changsha, P. R. China; 4 National Clinical Research Center for Geriatric Disorders, Changsha, P. R. China; 5 Department of Pharmaceutical Sciences, School of Pharmacy, University of Maryland at Baltimore, Baltimore, MD USA; 6 Zhengzhou Central Hospital Affiliated to Zhengzhou University, Zhengzhou University, Zhengzhou, P. R. China; 7 Shenzhen Center for Chronic Disease Control and Prevention, Shenzhen, P. R. China

**Keywords:** Cellular microbiology, Clinical microbiology

## Abstract

Vitamin B12 (VB12) deficiency, which may lead to hematologic and neurologic symptoms, has been associated with metformin use, but the underlying mechanism is unclear. Here we report the *B. ovatus* as an effective VB12 catcher which was enriched in the type 2 diabetes patients suffered from VB12 deficiency after 3 to 6 months of metformin treatment. Colonization of *B. ovatus* increased the plasma levels of methylmalonic acid and homocysteine in high-fat diet (HFD)-fed mice treated with metformin, and compromised the efficacy of metformin against the HFD-induced metabolic disorders. Mechanistically, metformin increased the intracellular accumulation of VB12 in *B. ovatus via btuB* upregulation and promoted ATP production for energy-dependent translocation of VB12 transporters at the inner membrane, leading to an enhanced colonization of *B. ovatus* to compete for VB12 with hosts and subsequently an aggravated VB12 deficiency in the host. Our findings illustrate a previously unappreciated mechanism of metformin leads to host VB12 deficiency by acting directly on gut bacteria to increase their VB12 uptake and consumption, and suggest that inter-host-microbe competition for nutrients may broadly impact human health and drug safety.

## Introduction

Metformin is the first-line glucose-lowering therapy for patients with type 2 diabetes and is recognized by all guidelines^1^. The main side effect listed on the package insert is an increased incidence of vitamin B12 (VB12) deficiency (between 5.8% and 33%)^2^, which is positively associated with the dosage and duration of metformin treatment.

VB12 (cobalamin), which is sourced from foods of animal origin, serves as an essential cofactor in DNA synthesis and fatty acid and amino acid metabolism. Clinical manifestations of VB12 deficiency include anemia, peripheral neuropathy, cognitive impairment, and depression^2,3^. Notably, diabetic peripheral neuropathy (DPN), which causes damage to nerves outside the brain and the spinal cord and increases the risk of developing Charcot foot, is one of the most common and serious complications of diabetes^4^. However, metformin-associated VB12 deficiency is normally ignored as an important etiology of neuropathy. In addition, pallor and mental symptoms, such as fatigue, and memory loss, can occur before frank anemia. Therefore, in diabetic patients, VB12 deficiency is usually underdiagnosed until more serious clinical concerns (e.g., irreversible neuropathy and dementia) occur, especially in elderly individuals^5^. A large-scale study (*n* = 1975) showed that compared to type 2 diabetes patients who received other antidiabetics, those who received metformin therapy had a higher prevalence of DPN, which was significantly associated with greater cumulative doses and a longer duration of metformin treatment, indicating that more attention should be given to the safety of long-term metformin treatment^6^. However, metformin-associated VB12 deficiency has not been well recognized in recent years, and the exact cause is still not known. Therefore, understanding the relationship between metformin use and VB12 status is clinically meaningful that could improve the efficacy of metformin and help prevent its side effects in patients with type 2 diabetes.

It has been suggested that metformin inhibits the Ca2+-dependent binding of the VB12-intrinsic factor (IF) complex, which impairs the absorption of VB12 in the terminal ileum^7,8^. However, it remains unknown why metformin-associated VB12 deficiency is characterized by high heterogeneity, according to a reporting of a previous meta-analysis^2^. Moreover, although an increased intake of calcium could help reverse the holotranscobalamin reduction induced by metformin, the serum total VB12 level did not effectively increase after one month of calcium carbonate supplementation^8^. Taken together, this shows that the mechanism by which metformin reduces serum concentrations of VB12 remains unclear.

Only 20% of bacterial and archaeal species, such as *Propionibacteriu*m *UF1*^9^, have been reported to be able to synthesize cobalamin upon complete, de novo, biosynthesis pathways^10^, resulting in a network of microbial interactions regarding cobamide sharing ranging from cross-feeding to competition^11,12^. Because of the prevalence of auxotrophy, microbes must rely on VB12-producing members to maintain their function. Therefore, representing a double-edged sword, the gut microbes not only provide VB12 for host humans through fermentation of complex carbohydrates but also compete with human for VB12. An excessive number of bacteria colonizing the small intestine could induce small intestinal bacterial overgrowth (SIBO), which is associated with the risk of type 2 diabetes^13^ due to slow small intestinal transit and increased consumption of the vitamins and nutrients needed by humans. However, few studies have evaluated a specific relationship between the gut microbes and metformin-induced VB12 deficiency.

Therefore, the primary aim of the present study was to investigate associations between gut microbiota and VB12 status in patients with diabetes on metformin. We hypothesized that some special strains could hijack VB12 from gastrointestinal tract aiding by metformin. Given that we analyzed bacterial electron transport chains changes under metformin, the secondary aim of our study was to explore potential mechanism by which the antidiabetic drug metformin acts on bacteria directly to modulate gut microbiota profile. We report that the relative abundance of *Bacteroides ovatus* in the gut was associated with metformin-induced vitamin B12 deficiency with data from a prospective patient cohort that included subjects with newly diagnosed type 2 diabetes treated with metformin for 3 to 6 months. We found that metformin treatment upregulated the expression of the microbial *btuB* gene and regulated adenosine triphosphate (ATP) production in *B. ovatus*, which strengthened its capacity to capture VB12 and subsequently reduced the serum levels of VB12 in the host.

## Results

### *B. ovatus* abundance was negatively correlated with the serum levels of VB12 in type 2 diabetes patients who received metformin treatment

**Fig. 1:**
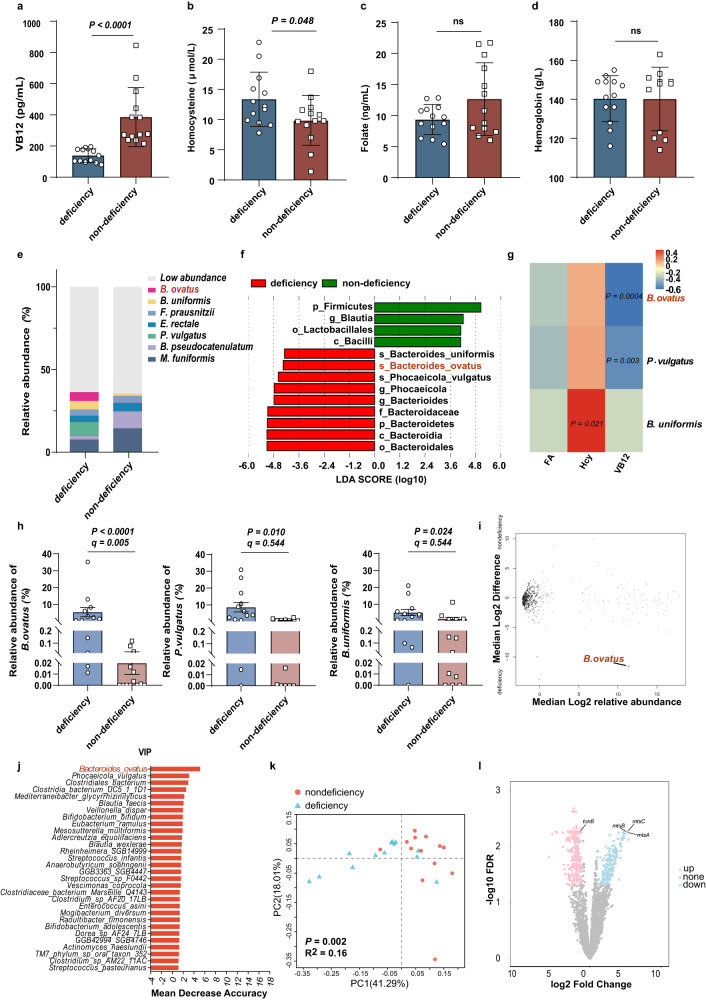
Metformin treatment induces different gut microbiota and vitamin B12 status between deficiency (*n* = 13) and non-deficiency (*n* = 13). **a** Serum total VB12 levels of type 2 diabetes patients after metformin treatment for 12–24 weeks. **b** The Homocysteine levels of type 2 diabetes patients after metformin treatment. **c** The folate levels of type 2 diabetes patients after metformin treatment. **d** The hemoglobin levels of type 2 diabetes patients after metformin treatment. **e** Stacked bar plots showing the average percent relative abundances at the genus level for deficiency and non-deficiency groups, respectively. The most abundant taxa are shown as differently colored bars, with lower abundance taxa grouped as a gray bar (Others). **f** Differences in bacterial taxonomy were ranked according to the linear discriminant analysis (LDA) effect size between deficiency and non-deficiency groups. **g** *Spearman’s* correlation between bacterial abundances and VB12 status biomarkers. **h** Abundance of *B. ovatus, P. vulgatus* and *B. uniformis* based on metagenomics data. Statistical significance is based on *P* values and *q* values obtained from MaAsLin2 analysis. **i** Volcano plot of ALDEx2 differential abundance testing on species in stool microbiotas of deficiency and non-deficiency subjects, with significantly different species highlighted (FDR < 0.05). **j** Variable Importance in Projection (VIP) of random forest classifier which was developed utilizing taxonomy-related data. **k** PCoA of KEGG ortholog (KOs) abundances; The deficiency group is shown in blue, and the non-deficiency group is shown in red; PERMANOVA test with the *Bray-Curtis* distance was used to assess the significant difference between the two groups, and the result showed significant separation of the deficiency and non-deficiency groups (*P* = 0.002). **l** Volcano p**l**ot of metagenomic sequencing data based on KEGG ortholog (KOs) in the deficiency and non-deficiency groups. *P* value determined by the Two-tailed unpaired Student’s *t* test (**b**, **d**) and Mann–Whitney *U* tests (**a**, **c**, **g**). All data are presented as mean ± SEM or mean ± SD.

It was reported that there was a statistically significant reduction in VB12 levels beyond a 6-week period of metformin therapy^14^. Herein, we designed a prospective clinical trial involving 12 to 24 weeks of metformin treatment in newly diagnosed type 2 diabetes patients. According to the well-established criteria^15^, we assessed VB12 status and the clinical manifestations of VB12 deficiency in 26 patients who were divided into the VB12 deficient (*n* = 13, serum total VB12 < 200 pg/mL) and nondeficient (*n* = 13, serum total VB12 > 200 pg/mL) groups. Their clinical characteristics are shown in Supplementary Table 1. The deficient patients had a significantly lower level of serum total VB12 and a higher level of homocysteine when compared with non-deficiency patients, but no significant differences in their folate and hemoglobin (Fig. 1a–d).

To investigate whether the gut microbiota was different between the deficient and nondeficient groups, we first performed whole-genome shotgun sequencing to explore the microbiome taxonomic identification via MetaPhlAn 4.0.6. Principal coordinate analysis (PCoA) revealed that the gut microbiota profile presented a clear alteration between the two groups (Supplementary Fig. 1a). In patients with VB12 deficiency, the relative abundance of Bacteroidetes was upregulated, while the relative abundance of Firmicutes was downregulated at the phylum level. At the genus level, the relative abundances of *Megamonas spp*., *Bifidobacterium spp*. and *Blautia spp*. were decreased, while the abundances of *Bacterioides spp*., *Phocaeicoia spp*. and *Roseburia spp*. were enhanced (Supplementary Fig. 1b, c). Consistent with the species level (Fig. 1e), analysis with the linear discriminant analysis (LDA) effect size (LEfSe) method revealed that the deficiency group was characterized by *B. ovatus*, *B. uniformis*, and *Phocaeicola vulgatus* (Fig. 1f). And the richness of *B. ovatus* and *P. vulgatus* were negatively correlated with the VB12 level (Fig. 1g). Differential analyses were performed using the MaAsLin2, ALDEx2 and Wilcoxon rank-sum test with adjustment for covariates. We found that *B. ovatus* presented significantly higher abundance in deficiency group (Fig. 1h, i; Supplementary Fig. 1d). Next, we used a random forest classifier to select the discriminating features in the gut microbiota for differentiating species included *B. ovatus* and *P. vulgatus* (Fig. 1j).

Gut microbiota functional gene profiles at the KO level were also different between the two groups (Fig. 1k). TonB-dependent transporters (TBDTs) in the gram-negative bacterial outer membrane (OM) bind and transport environmental VB12 across the OM into the periplasmic space^16,17^. The *TonB* gene, which encodes the TonB protein, was enriched in the deficient group. In contrast, the expression of *mtsA*, *mtsB*, and *mtsC*, which encode iron/zinc/manganese/copper transport system substrate-binding proteins, was relatively low in the deficient group (Fig. 1l). *Bacteroides spp*. biosynthesis B12-dependent enzymes. It was reported *P. vulgatus* could code VB12 biosynthetic genes (such as *CbiA*, *CbiC*)^18^, but not *B. ovatus* which must capture VB12 from the digestive tract^19^. These results suggest that the capacity for VB12 uptake by certain gut microbes, such as enriched *B. ovatus*, might be increased under metformin treatment.

### Metformin increased the uptake of VB12 in *B. ovatus* via *btuB* upregulation

**Fig. 2:**
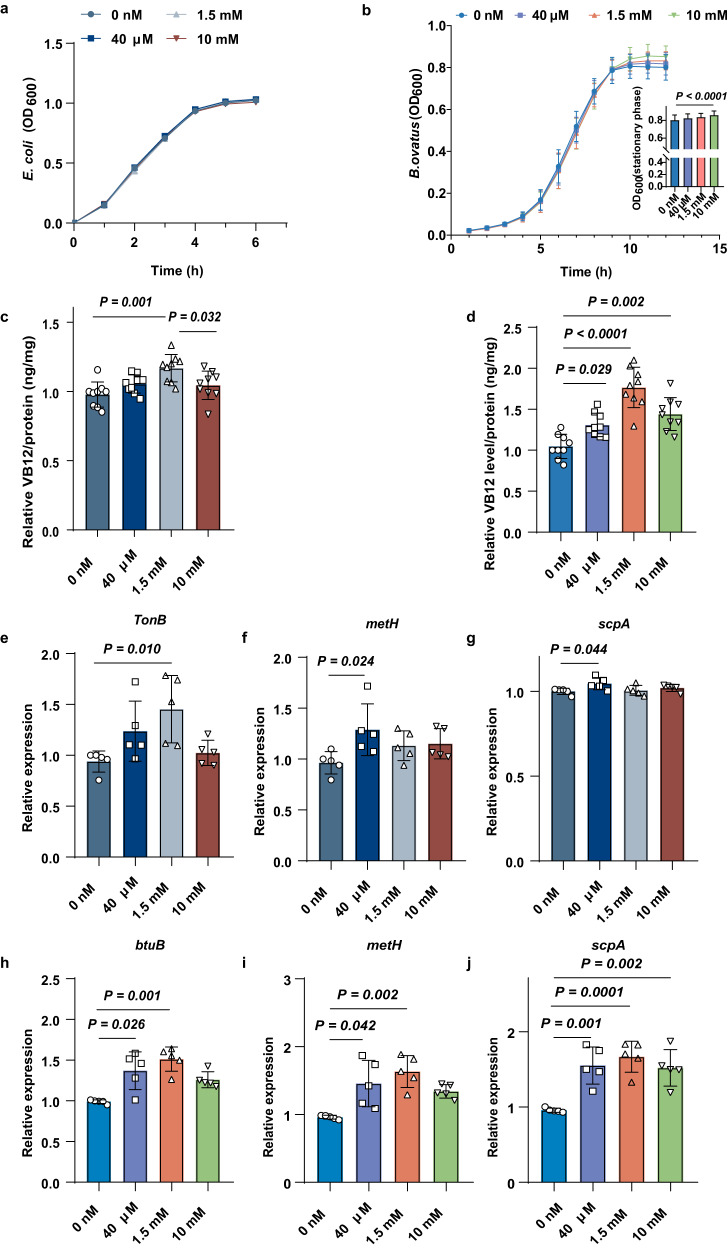
Metformin increased VB12 concentrations inside E. coli or B. ovatus via TonB or btuB upregulation, respectively. **a**, **b** Growth curves of *E. coli* or *B. ovatus* with metformin at different concentrations (3 technical replicates of 6 biological replicates for each group). **c**, **d** VB12 concentrations inside *E. coli* or *B. ovatus* with metformin at different concentrations (*n* = 9). **e**–**g** Relative mRNA abundances of *TonB*, *metH* and *scpA* in *E. coli* treated with metformin for 1 h (*n* = 5). **h**–**j** Relative mRNA abundances of *btuB*, *metH* and *scpA* in *B. ovatus* treated with metformin for 7 h (*n* = 5). *P* value determined by the Kruskal–Wallis test (**b**, **e**–**h**, **i**) and the one-way ANOVA with Tukey’s or Dunnett’s tests (**c**, **d**, **f**, **g**, **j**). All data are presented as mean ± SEM or mean ± SD.

We studied the effect of metformin treatment on the accumulation of VB12 in *B. ovatus*. The concentration of VB12 in the bacterial pellet was determined by using LC‒MS/MS (Supplementary Fig. 2a–e). The current knowledge about TBDTs in bacteria is limited, except in *Escherichia coli*^20^. As a control, we used *E. coli K12*, which appears to take up a high level of VB12 from the growth media. While no significant change was found in the growth curves of *E. coli* treated with a range of metformin concentrations (Fig. 2a), metformin treatment dose-dependently promoted the growth of *B. ovatus* in the stationary phase via promoting its adaptability (Fig. 2b). We then examined the effect of metformin treatment on the intracellular VB12 concentration for the two strains growing in minimal media supplemented with cyanocobalamin. We chose a concentration of 3.7 nM for cyanocobalamin^19^ because it can not repress the RNA elements associated with B12 riboswitches that can change conformation upon specific binding to VB12. Notably, metformin significantly increased the intracellular accumulation of VB12 in both *E. coli* and *B. ovatus* (Fig. 2c, d).

TBDTs consist of TonB, ExbB, and ExbD, which are energized by the electrochemical gradient^17^. The corrinoid transport loci in both *E. coli* and *Bacteroides spp*. have an OM transporter (BtuB) to capture environmental VB12, and then a periplasmic binding protein (BtuF) and inner membrane (IM) ATP-dependent transporters (BtuC and BtuD) to transfer VB12 from the periplasm to the cytoplasm^21^. Consistent with its effect on intracellular VB12 concentrations, metformin (1.5 mM) significantly upregulated the expression of *TonB* in *E. coli* in vitro (Fig. 2e), but no significant changes were found for the expression of genes encoding other TBDTs or binding proteins (Supplementary Fig. 3a). The mRNA level of *btuB* in *B. ovatu*s was much higher after metformin incubation for 7 h (40 μM, 1.5 mM) (Fig. 2h). Due to the riboswitch translational control of *btuB* expression at the 5′ end^16^, we hypothesized that the expression of *btuB* might eventually be inhibited following the gradual intracellular accumulation of VB12. Consistent with this hypothesis, we found that the expression of *btuB* appeared to rise in a dose dependent manner at 12 h (Supplementary Fig. 3b), followed by an abrupt decline at 24 h after metformin treatment (Supplementary Fig. 3e).

Vitamin B12 (cobalamin) serves as a cofactor for both *metH* and *scpA*, which catalyze the formation of methionine *via* the transfer of a methyl group from 5-methyl-tetrahydrofolate to homocysteine and the isomerization of methylmalonyl-CoA to succinyl-CoA, respectively. To test whether VB12 was transported across the OM and IM into the cytoplasm, we chose the expression of *metH* and *scpA* as biosensors to represent the intracellular level of available VB12^17^. Interestingly, the mRNA levels of *metH* and *scpA* in *B. ovatus* were consistent with that of *btuB* (Fig. 2i, j, Supplementary Fig. 3c, d, Supplementary Fig. 3f, g), which implied that metformin treatment increased the capability of *B. ovatus* to compete for VB12. However, only moderate expression changes in *metH* and *scpA* were found in *E. coli* treated with 40 μM metformin but not in those treated with 1.5 mM (Fig. 2f, g). These data indicated that metformin could enhance microbial VB12 uptake. However, not all VB12 can be effectively used by different bacteria.

### Metformin treatment could alter bacterial ATP production via its effects on the electron transport chain

**Fig. 3:**
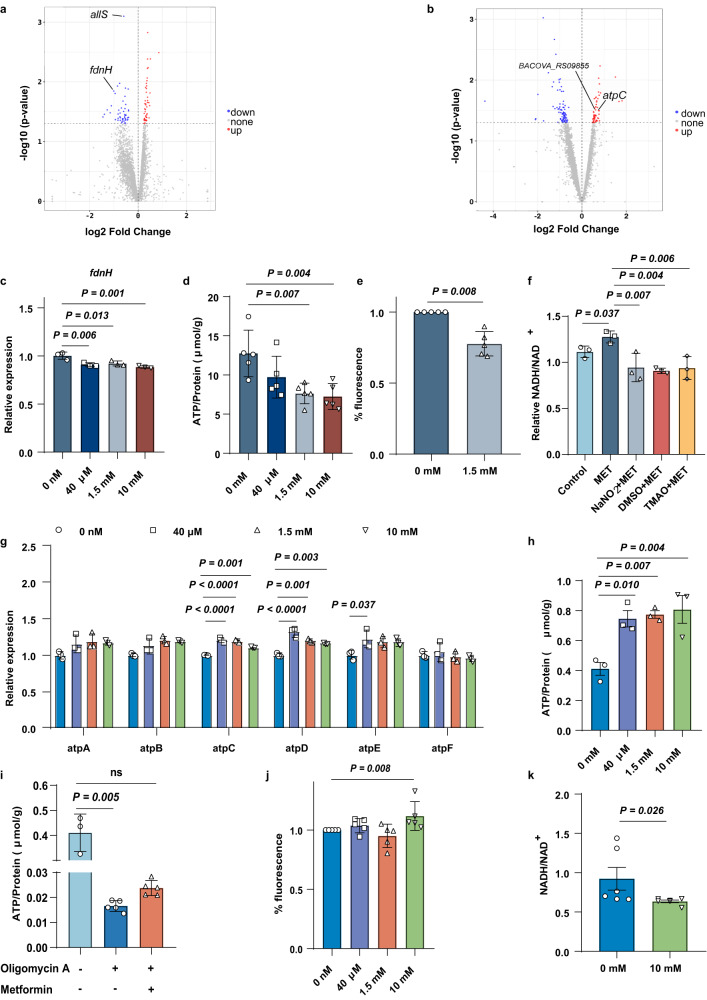
Metformin regulates the bacterial electron transport chains. **a** Volcano plot showing differential expression of the 97 genes of *E. coli* treated with metformin (at 1.5 mM) compared to control. The blue dots represent genes significantly down-regulated, and red dots represent up-regulated genes (*n* = 3). **b** Volcano plot showing differential expression of the 80 genes of *B. ovatus* treated with 1.5 mM metformin compared to control. The blue dots represent genes significantly down-regulated, and red dots represent up-regulated genes (*n* = 3). **c** Relative mRNA abundances of *fdnH* in *E. coli* treated with metformin for 1 h (*n* = 3). **d** The concentrations of ATP in *E. coli* treated with metformin for 1 h (*n* = 5). **e** The membrane potential of *E. coli* treated with metformin for 1 h (*n* = 5). **f** The NADH/NAD^+^ ratio of *E. coli* treated with metformin and/or terminal electron acceptor (DMSO, NaNO_2_ and TMAO) for 1 h (*n* = 3). **g** Relative mRNA expression of encoding enzymes involved in ATP synthase genes in *B. ovatus* treated with metformin for 7 h (*n* = 3). **h** The concentrations of ATP in *B. ovatus* treated with metformin for 7 h (*n* = 3). **i** The concentrations of ATP in *B. ovatus* treated with 1.5 mM metformin for 7 h and 2 μM oligomycin A for 1 h (*n* = 3-5). **j** The membrane potential of *B. ovatus* treated with metformin for 7 h (*n* = 5). **k** The NADH/NAD^+^ ratio of *B. ovatus* treated with 10 mM metformin for 7 h (*n* = 6). *P* value determined by the one-way ANOVA with Tukey’s or Dunnett’s tests (**c**, **d**, **f**–**h**), the Kruskal–Wallis test (**i**) and Mann–Whitney *U* test (**e**, **j**, **b**). All data are presented as mean ± SEM or mean ± SD.

We performed bacterial RNA sequencing to identify the biological processes that were affected by metformin treatment. The volcano plot highlights the differentially expressed genes of *E. coli* (49 genes were upregulated; 48 genes were downregulated) and *B. ovatus* (46 genes were upregulated; 34 genes were downregulated) following metformin treatment (Fig. 3a, b).

The *E. coli fdnH* gene, which encodes a subunit of formate dehydrogenase that is involved in NAD-dependent anaerobic nitrate respiration, was downregulated (Fig. 3c). Through pathway analysis, the sequencing data indicated that metformin treatment led to decreased transmembrane transporter activity/vitamin binding GO functions and downregulation of the selenocompound metabolism KEGG pathway, suggesting that ATPase function and the methionine biosynthetic pathway were altered with metformin treatment (Supplementary Fig. 4a, b). Unlike TBDTs, which are driven by a proton motive force, the IM protein BtuCD is an ATP-binding cassette (ABC) transporter that pumps compounds across the phospholipid bilayer and is triggered by the hydrolysis of ATP^22^. Metformin can inhibit respiratory complex I to decrease ATP synthesis in mammals. Therefore, to test whether metformin would also disturb the bacterial electron transport chains in ATP synthesis, we then detected intracellular ATP production. As expected, metformin treatment (1.5 mM and 10 mM) was found to reduce ATP levels in *E. coli* (Fig. 3d). Respiratory chain complexes (complexes I, III, and IV) are proton pumps that maintain the electrochemical proton gradient, which is exploited by ATP synthase to produce ATP from ADP in the generation of cellular energy^23^. Consistently, the *E. coli* membrane potential was suppressed by metformin treatment, as determined from the bacterial membrane potential assay (Fig. 3e). Bacterial complex I plays an important role in maintaining the proton electrochemical potential, wherein NADH is oxidized to NAD^+^ along with H^+^ dissociation to create the proton motive force^24^. We found that the NADH/NAD^+^ ratio was significantly higher in *E. coli* after treatment with 1.5 mM metformin, which might contribute to the compromised membrane gradient (Fig. 3f). A high-energy electron is passed along an electron transport chain to electron acceptors. Dimethyl sulfoxide (DMSO), trimethylamine N-oxide (TMAO), and nitrate can be used by *E. coli* anaerobic respiration as the terminal electron acceptors^25^. As Fig. 3f shown, the elevated NADH/NAD^+^ ratio induced by metformin treatment was abolished during anaerobic growth with the addition of these terminal electron acceptors.

In contrast, the sequencing data indicated that metformin treatment led to increased ferric iron binding GO functions and upregulation of the ribosome KEGG pathway, suggesting that metal ion transport and bacterial growth were promoted with metformin treatment in *B. ovatus* (Supplementary Fig. 4c, d). Moreover, we found that the expression of not only *atpC* but also other genes encoding ATP synthases (e.g., *atpD* and *atpE*) was increased by metformin treatment in *B. ovatus* (Fig. 3g). Moreover, as expected, metformin treatment increased the intracellular level of ATP in *B. ovatus* (Fig. 3h). Oligomycin A, as an inhibitor of ATP synthases, greatly reduced the ATP level, which could be slightly reversed by metformin (1.5 mM) (Fig. 3i). For complex I, only a high concentration of metformin (10 mM) could increase the membrane potential and decrease the NADH/NAD^+^ ratio (Fig. 3j, k), indicating that metformin treatment enhanced ATP production in *B. ovatus* primarily through its effect on complex V, instead of complex I, in the respiratory chain.

Altogether, these data suggest that metformin could alter the bacterial the electron transport chain via complex I or V to influence ATP synthesis.

### *B. ovatus* accelerated metformin-induced VB12 deficiency in HFD-induced mice

**Fig. 4:**
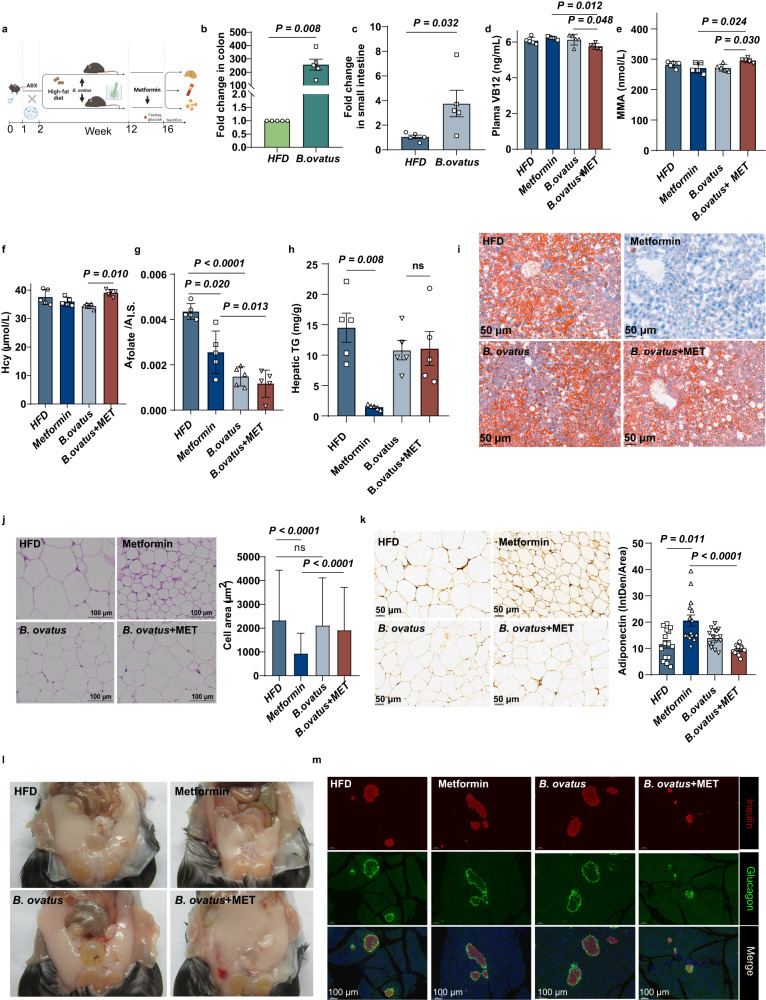
Colonization of B. ovatus accelerate metformin-induced VB12 deficiency in HFD-induced mice. **a** Introduction of *B. ovatus* strain in microbiota-depleted mice (*n* = 5 mice/group). **b**, **c** Fold changes of relative abundance of *B. ovatus* in colon contents and small intestine contents of mouse recipients based on q-PCR analysis, respectively (*n* = 5). **d** The concentrations of plasma VB12 (*n* = 5). **e** The concentrations of plasma methylmalonic acid (MMA) (*n* = 5). **f** The concentrations of plasma homocysteine (Hcy) (*n* = 5). **g** The relative levels of plasma folate (*n* = 5). **h** Hepatic TG levels (*n* = 5). **i** Oil Red O staining on liver tissues; scale bars, 50 μm (*n* = 3). **j** H&E staining of subcutaneous fat (sWAT); scale bars, 100 μm (*n* = 3). **k** Adiponectin immunohistologic staining of sWAT sections; scale bars, 50 μm (*n* = 3). **l** Representative gross the peri-gonadal fat images from HFD and *B.ovatus*-colonized type 2 diabetes mice treated with vehicle or metformin for 4 weeks. **m** Sections of mouse pancreas were analyzed by immunofluorescence using antibodies to Insulin (red) and glucagon (green); scale bars, 100 μm (*n* = 3). *P* value determined by the Mann–Whitney *U* test (**b**, **c**, **h**), one-way ANOVA with Tukey’s or Dunnett’s tests (**d**, **e**, **g**) and Kruskal–Wallis test (**f**). All data are presented as mean ± SEM or mean ± SD.

To further validate the influence of *B. ovatus* abundance on the effect of metformin treatment on VB12 disposition, we designed in vivo animal studies (Fig. 4a). The colonization of *B. ovatus* in microbiota-depleted mice was conducted. After treatment with antibiotics followed by *B. ovatus* transplantation for ten weeks in mice fed a high-fat diet, the abundance of *B. ovatus* was significantly enhanced in both the colon (Fig. 4b) and small intestine (Fig. 4c). *Via* complexation with an IF, VB12 can be internalized into the ileal cell where the complex is degraded within lysosomes to release VB12 and then VB12 is exported into the portal circulation^15^. And BtuG, a protein on the surface of *Bacteroides*, could remove VB12 from IFs with high affinity and couple with btuFCD for VB12 uptake^19^. Therefore, colonizing the small intestine, *B. ovatus* would be expected to compete with the human host for VB12 uptake. In animal cells, VB12 assists methionine synthase in the catalysis of the methylation of homocysteine to methionine or aids methylmalonyl-CoA mutase for mitochondrial conversion of methylmalonyl-CoA to succinyl-CoA^15^. Therefore, a low level of VB12 could inhibit these biochemical reactions, leading to cellular accumulation of homocysteine (Hcy) or methylmalonic acid (MMA) and subsequently an increased risk of complications (e.g., hyperhomocysteinemia). After metformin treatment for four weeks, the total serum VB12 concentration was lower in the mice with gut *B. ovatus* implantation than in those that did not receive metformin treatment, and the serum concentration of MMA, a typical blood biomarker of VB12 homeostasis, was significantly higher (Fig. 4d, e). Consistently, the Hcy levels of *B. ovatus*-colonized mice were elevated by metformin treatment as well. In addition, the folate level was significantly reduced after metformin treatment and/or *B. ovatus* colonization (Fig. 4f, g).

Choline could be produced from the newly formed phosphatidylcholine in the liver. The biochemical reaction requires S-adenosylmethionine as the methyl donor, which is regulated by the levels of VB12 and folate, reflecting the cellular methylation capacity^26^. Therefore, a low level of VB12 or folate in the liver may reduce choline availability, leading to dysregulation of cholesterol metabolism and hepatic steatosis. Metformin treatment significantly reduced fat deposition in HFD-fed mice. We found that the efficacy of metformin against HFD-induced fat deposits in the liver was significantly compromised by *B. ovatus* colonization (Fig. 4h, i). Furthermore, while metformin treatment could reduce the size of adipocytes in fat tissues, the effect was abolished in mice with gut *B. ovatus* implantation. Consistently, the beneficial increase in adiponectin expression in fat tissues after metformin treatment in HFD-fed mice was not observed in the mice with gut *B. ovatus* implantation (Fig. 4j–l). Folic acid (FA) and VB12 can prevent pancreatic beta cells from failing by suppressing oxidative stress^27^. Immunohistochemistry and stereology examination showed that the islet volume was not affected by metformin treatment alone or gut *B. ovatus* implantation alone in HFD-fed mice; however, the islet volume was much smaller in the HFD-fed mice received both metformin treatment and gut *B. ovatus* implantation, along with a reduced number of beta cells that were interspersed among an increased number of alpha cells (Fig. 4m).

**Fig. 5:**
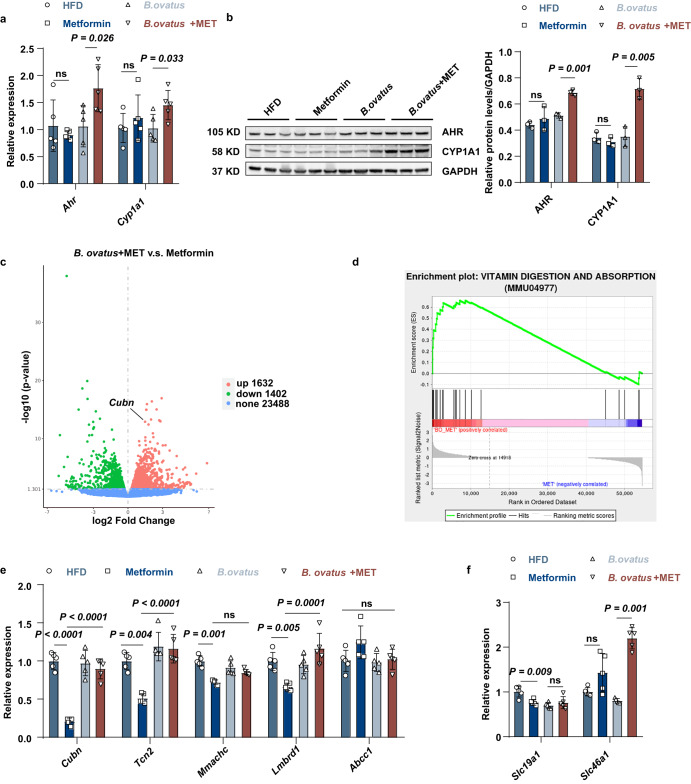
Low VB12 status induced AhR transcriptional activity and no significant change in VB12 absorption pathway in B. ovatus- colonized mice. **a** Relative mRNA abundances of *Ahr*, its target gene *Cyp1a1* in the liver (*n* = 5). **b** Western blot analysis (cropping of blot images) of hepatic AHR and CYP1A1 protein expression and the statistical graph (*n* = 3). **c** Volcano plot of significantly upregulated (red), downregulated (green) genes and non-significant (blue) genes in *B. ovatus* + MET and metformin group mice (*n* = 3). **d** Enrichment plots of vitamin digestion and absorption pathway enriched in Gene set enrichment analysis (GSEA), showing the profile of the running ES Score and positions of gene set members on the rank-ordered list (*n* = 3). **e** Relative mRNA levels of VB12 absorption and transport genes (*n* = 5). **f** Relative mRNA expression of folic acid absorption genes (*n* = 5). *P* value determined by the Two-tailed unpaired Student’s *t* test (**a**, **b**), one-way ANOVA with Tukey’s tests (**e**, **f**) and Kruskal–Wallis test (**e**, **f**). All data are presented as mean ± SEM or mean ± SD.

In addition, VB12 and FA have been reported as natural antagonists of the aryl hydrocarbon receptor (AhR), which can block AhR nuclear localization^28^. We found that metformin administration in *B. ovatus*-colonized mice could induce the relative expression of AhR and its downstream target CYP1A1 in the liver which was not detected in the HFD-fed mice that received metformin treatment alone or gut *B. ovatus* implantation alone (Fig. 5a, b).

To further reveal alterations in cobalamin absorption in host mice, we performed RNA-seq analysis with ileum tissues. A total of 3034 differentially expressed genes (DEGs) were identified between the HFD-fed mice that received gut *B. ovatus* implantation alone and those additionally received metformin treatment (Fig. 5c). These genes, including those encoding the IF-VB12 receptor complex (*Cubn*), were found to be enriched in vitamin digestion and absorption pathways in KEGG pathway analysis (Fig. 5d and Supplementary Fig. 5a). Moreover, the sequencing data also indicated that *B. ovatus* + metformin treatment led to increased metabolism of water-soluble vitamins Reactome pathway and upregulation of the cofactor binding GO function (Supplementary Fig. 5b, c). We further examined the relative expression of the genes involved in IF-VB12 uptake/degradation and VB12 efflux/transport. No significant changes were found between the HFD-fed mice received gut *B. ovatus* implantation alone and those additionally received metformin treatment; however, the expression of these genes was overall lower in the HFD-fed mice that received metformin treatment alone (Fig. 5e). The *Slc46a1* gene encodes a transmembrane proton-coupled folate transporter protein that has been reported to be increased in folate-deficient mice^29^. The expression of *Slc46a1* was significantly upregulated in the *B. ovatus* + MET group compared to the *B. ovatus* group, but not *Slc19a1* (Fig. 5f, Supplementary Fig. 5d). Overall, these data suggest that enrichment of *B. ovatus* in the gut may aggravate metformin-induced VB12 deficiency by capturing cobalamin instead of disturbing the absorption of VB12.

## Discussion

Clinical manifestations of VB12 deficiency, such as classical megaloblastic anemia, hyperhomocysteinaemia, peripheral neuropathy and cognitive deficits, may reduce the potential benefit of metformin. Although there are classic blood biomarkers (total VB12, holotranscobalamin, Hcy, and MMA), diagnosing VB12 deficiency is difficult due to its association with other diseases, such as autoimmune disorders, renal insufficiency and Alzheimer’s disease^15^. A variety of factors could contribute to VB12 deficiency, such as a lack of VB12-containing food consumption, a reduction in gastric acid production, alterations in calcium-dependent membrane function, and the presence of SIBO, intrinsic factor deficiency, and lysosomal dysfunction. VB12 is only synthesized by certain bacteria and archaea and is available from animals or fish as a result of microbial-host interactions^15^. While plants do not contain VB12, type 2 diabetes patients are usually advised to increase their intake of fruits, vegetables, whole grains, and legumes and to decrease their consumption of red meat, saturated fats, and high-carbohydrate foods. Some patients even choose to become vegetarians, which limits their food sources of VB12 and may also alter their gut microbiota composition. Although the microbiota plays an important role in contributing essential cofactors to the host, microbial sources of VB12 usually accumulate in herbivorous ruminant animals rather than humans. Moreover, the gut microbiota could convert dietary VB12 into alternate corrinoids that are difficult for humans to use directly^19^. Therefore, the gut microbiota, which lacks vitamin biosynthetic pathways, may interfere with host VB12 absorption. However, the obstacle posed by the microbiota to the host’s absorption of VB12 has yet to be confirmed, particularly in the context of host drug therapy.

*B. ovatus* is a common human gut bacterium with various polysaccharide-utilizing loci^30,31^ can degrade and grow on complex plant-based dietary fibers. It expresses corrinoid transport-encoding conjugative transposons, which are mobile and cause the exchange of genes required for the transport of corrinoids to enable colonization^21^. Our data show that *B. ovatus* could colonize the small intestine to transport VB12 and aggravate metformin-induced VB12 deficiency in the host.

**Fig. 6:**
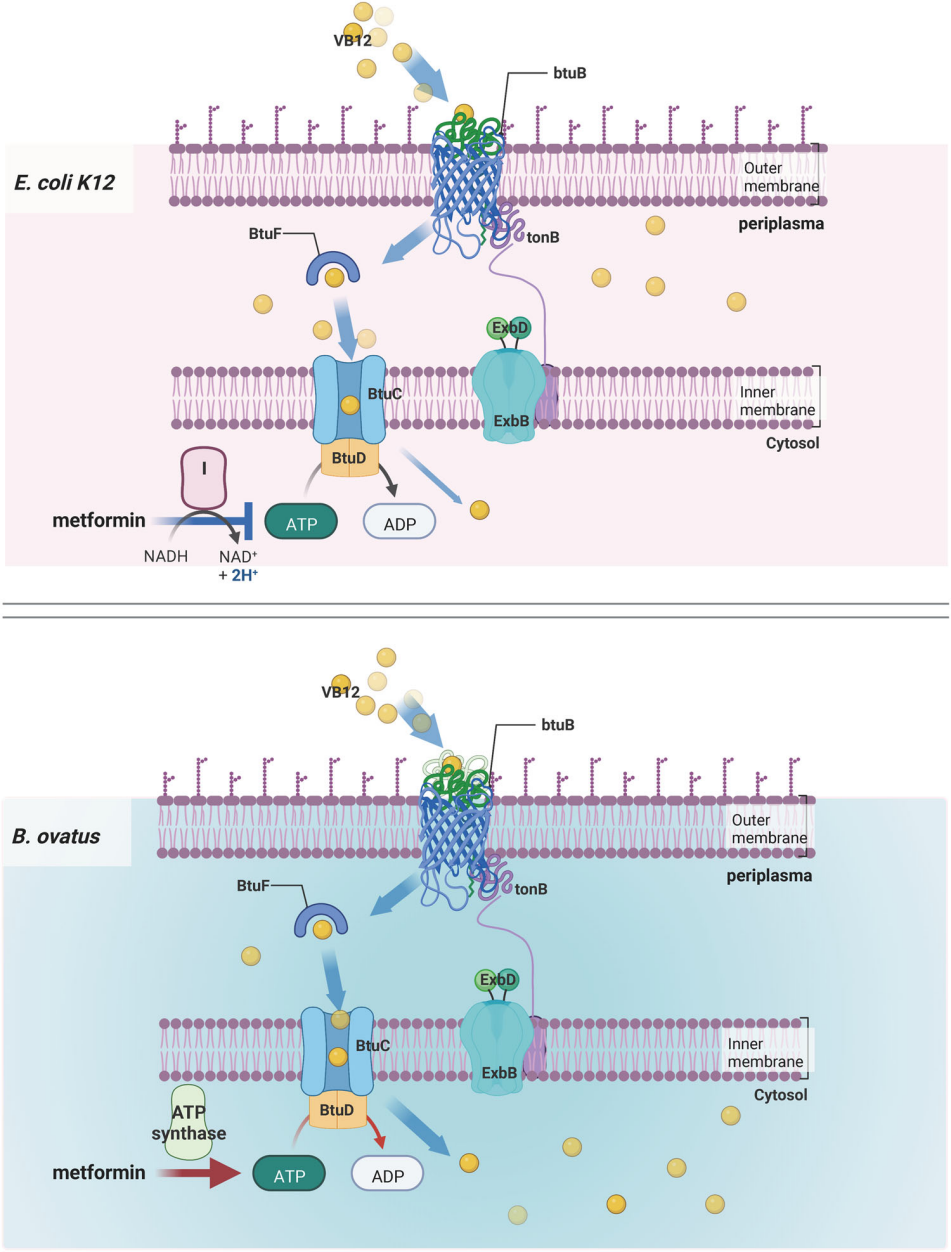
Metformin inhibits the *E. coli* respiratory chain complex I, and VB12 is blocked and accumulated in the periplasm. By contrast, metformin upregulates the *B. ovatus* respiratory chain complex V, and VB12 could be transported across inner membrane to cytoplasm. The diagram was created using BioRender.

Metformin use has been associated with changes in the human gut microbiome composition and could alter the expression of the genes encoding metalloproteins or metal transporters in the gut microbiota of type 2 diabetes patients^32,33^. While metformin is not an antibiotic drug, it can inhibit the growth of some bacteria, such as *B. fragilis*^34^ and *E. coli OP50*^35^, for which metformin treatment may lead to increased levels of 5-methyl-tetrahydrofolate and decreased levels of methionine. Metformin treatment may thus interfere with the one-carbon metabolism of the bacteria, which is dependent on VB12 as a cofactor. We observed that metformin was able to increase the cellular content of VB12 in these bacteria *via* upregulation of *TonB* or *btuB* expression. However, the VB12 that is captured by IM proteins may not pass although the inner cytoplasmic cell membrane because the activity of IM ABC-transporter proteins, which is energized by ATP hydrolysis, could be influenced by metformin. We found that metformin could inhibit the electron transport chain in *E. coli*, leading to decreased ATP production. Thus, the elevated VB12 concentration and upregulated *TonB* did not cause increased expression of *metH* and *scpA* due to ATP-deficient ExbBD. However, because metformin could upregulate ATP synthase in *B. ovatus*, the VB12 captured by BtuB in this strain could be continuously transported across the periplasm and then into the cytoplasm (Fig. 6). Indeed, in addition to cobalamin, other substrates, such as iron complexes, nickel chelates, and carbohydrates, could also be imported by TonB-dependent transporters^16^. These substrates are important for maintaining bacterial growth and human health because they facilitate the conversion of chemical nutrients into biomass. Therefore, our findings provide new insights into the effect of metformin on the interaction between the gut microbiota and hosts.

We showed that VB12 deficiency was more easily induced by metformin in *B. ovatus*-colonized mice, but no in *B. ovatus* alone mice. Interestingly, while the absorption of VB12 could be significantly inhibited by metformin in HFD-fed mice, the absorption was not affected in the mice that received both *B. ovatus* implantation and metformin treatment. The downregulation of gene expression related to several processes in the mice that received metformin treatment alone, including the complexation of VB12 with cubilin (*Cubn*), the transport of VB12 out of lysosomes (*Lmbrd1*), the conversion of VB12 into adenosylcobalamin and methylcobalamin (*Mmachc*), and the transport of VB12 from the bloodstream to cells (*Tcn2*), indicated that metformin may directly disturb the ileal absorption of VB12. In addition, metformin inhibited the activity of vacuolar-type ATPase (V-ATPase) *via* the PEN2-ATP6AP1 axis^36^. V-ATPase exerts a crucial function in lysosomal acidification to activate acid proteases that are essential for the degradation of IF and subsequent release of VB12 into the cytoplasm^15^. Thus, metformin-induced VB12 deficiency could result from lysosomal dysfunction. VB12 deficiency associated with long-term treatment with metformin has been observed in patients from different countries and of different ethnicities^37,38^, with a highly variable prevalence (between 5.8% and 33%)^2^. The high heterogeneity can hardly be explained by host factors alone. There are 500 to 1000 different microbial species residing in each person’s intestinal tract, and the gut microbiota is characterized by large interindividual variability regulated by genetic variation, maternal factors, nutrition, physiological state, drugs, and even social stress, which affect its ability to occupy and compete for limited space and resources. Therefore, the startling diversity of these taxonomic and functional commensal communities could work as mediating factors to influence the host VB12 status. *B. ovatus* was found in 70.053% of healthy people (*n* = 11,406, GMrepo). In our previous data, individuals with type 2 diabetes harbored unique relative abundances between 0% and 32.5% (*n* = 112) with high variation (1.31% ± 3.73%), suggesting that the complex cross-talk between *B. ovatus* and the host with respect to cobalamin could even be extended to other *Bacteroides spp*. Altogether, our findings highlight the importance of the gut bacteria, especially *B. ovatus*, in VB12 deficiency associated with metformin use.

In this study, we have identified *B. ovatus* as a mediator, which reduced plasma VB12 of host on metformin treatment. However, the exact domain structures of TonB-dependent transporters in the *B. ovatus* remains unknown. It was reported that the structural features of TBDTs *in B. thetaiotaomicron* with at least three distinct subtypes which regulates in different ways to tune nutrient uptake^39^. *B. ovatus* deploys multiple PUL to uptake different types of complex carbohydrates, and could promote the growth of *P. vulgatus*^40^. A critical question is whether *B. ovatus* also encode unique TBDTs to utilize nutrient, which remain to be determined.

In conclusion, type 2 diabetes patients with abundant *B. ovatus* in the gut may be more likely to suffer from VB12 deficiency when treated with metformin, which enhances the bacterial capacity for VB12 uptake. Metformin may act on the respiratory chain in different manners in the regulation of the bacterial life cycle and host–microbiota interactions. Therefore, routine testing of the serum levels of VB12 and homocysteine may be considered for type 2 diabetes patients treated with long-term metformin, especially *B. ovatus*-rich patients. The various safety issues of metformin should be given more attention considering the unique personal gut microbiota, especially the correlation of DPN with bacteria.

## Methods

### Human subjects

Subjects diagnosed with type 2 diabetes between April 2019 and June 2020 at Zhengzhou Central Hospital Affiliated to Zhengzhou University were recruited according to strict inclusion and exclusion criteria: Chinese patients with newly diagnosed T2DM were recruited for the study (aged 18–65 years; 18.5 kg/m^2^ < BMI < 35.0 kg/m^2^) and has never been treated with antidiabetic drugs before this study. The exclusion criteria were: (1) type 1 diabetes, gestational diabetes or other secondary diabetes; (2) continuous antibiotic or probiotics use for > 3 days within 3 months prior to enrollment; (3) a severe hypoglycemia event (blood glucose levels ≤ 3.9 mmol/L) of unknown cause occurred within 3 months before this study; (4) a past medical history of diabetic ketoacidosis, lactic acidosis or nonketotic hyperosmolar syndrome; (5) organ failure or clinically significant complications: severe hepatic diseases (including chronic persistent hepatitis, liver cirrhosis or the co-occurrence of positive hepatitis B virus surface antigen and abnormal hepatic transaminase (serum concentrations of alanine transaminase or aspartate transaminase >2.5× the upper normal limit); severe kidney disease (creatinine > 2 mg/dl); severe organic diseases, including cancer, coronary heart disease, myocardial infarction or cerebral apoplexy; infectious diseases, including pulmonary tuberculosis and HIV carrier; severe diabetic complications (diabetic retinopathy, diabetic neuropathy, diabetic nephropathy and diabetic foot); pituitary dysfunction; transplant recipient; gastrointestinal surgery (except for appendicitis or hernia surgery); severe mental illness within 6 months prior to enrolment; bleeding disease or bleeding tendency; systolic blood pressure ≥160 mmHg or diastolic blood pressure ≥100 mmHg; anemia (for men, less than 12.0 grams per deciliter; for women, less than 11.0 grams per deciliter); (6) immunologic endocrine disorders associated with high blood sugar; (7) continuous use of weight-loss drug for > 1 month; (8) alcoholism; (9) drug allergy and high sensitivity to environmental substances; (10) treatment with moderate and strong inducer or inhibitors of CYP3A4; (11) pregnancy, lactation, an intent to become pregnant during the course of the study.

Participants were administered with metformin hydrochloride treatment (1000 mg BID for 1–2 weeks, then 2000 mg per day, Sino-American Shanghai Squibb Pharmaceuticals Ltd.). Such treatment lasted 12–24 weeks, participants were assigned to the deficiency group (serum total VB12 < 200 pg/mL) or to the non-deficiency group (serum total VB12 > 200 pg/mL). Anthropometric measurements, biological samples, and metabolic testing were carried out at the end-of-intervention term. Peripheral blood samples were centrifuged at 3320 *g* and 4 °C for 10 min to obtain the plasma. The levels of blood biochemical indicators were measured using an autoanalyzer. Feces were collected and stored at −80 °C until analysis.

### Human gut microbiota DNA extraction and metagenomics sequencing

Human fecal genomic DNA was extracted using a Magnetic Soil and Stool DNA kit (TIANGEN Biotech) and sequenced on the illumina novaseq platform, and 150 bp paired-end reads were generated. After removal of low-quality reads (with a quality score < 20), sequencing adapters and reads shorter than 80 bp using Trim Galore, we obtained on average 72.6 million (range 65.9 to 86.2 million) high-quality clean reads for each fecal sample. Clean reads were mapped against the Human Reference Genome (http://hgdownload.soe.ucsc.edu/goldenPath/hg38/bigZips/) using Bowtie2 (version 2.3.1, default parameters). The unmapped reads were assembled using the metaSPAdes (SPAdes-3.13.1, k-mer sizes: 21, 33, 55, 77) and controlled by QUAST. Then, the contigs longer than 500 bp were used to predict genes with MetaGeneMark (GeneMark.hmm version 3.38). A non-redundant gene catalog was constructed with CD-HIT with a sequence identity cutoff of 0.9 and a minimum coverage cutoff of 0.9 for the shorter sequences. The abundance of genes in each sample was calculated by mapping the non-redundant gene catalog, counting the number of aligned reads (samtools depth, default parameters) and normalizing by gene length, then were transformed to relative abundance by (10000000 × (x/sum(x))). Taxonomic profiling of metagenomic samples was performed using MetaPhlAn 4.0.6 (with reference database mpa_vOct22_CHOCOPhlAnSGB_202212, included bacterial, archaeal and fungal taxonomic classification)^41^. The functional annotations of newly assembled genes were performed using KOBAS (version 3.0, default parameters) against the KEGG (http://kobas.cbi.pku.edu.cn/). The abundance of a KEGG orthology/module was calculated by summing the abundances of genes annotated to the same feature. Downstream analyses were conducted in the R (version 3.5.1; 4.2.0) using multiple packages, including *reshape2*, *vegan*, *ggplot2*, *Maaslin2* and *ALDEx2*. Beta diversity was calculated based on *Bray-Curtis* distances (normalized data) and distance matrix used to perform an ordination principal coordinate analysis (PCoA). The LEfSe analysis was performed using Galaxy^42^. Bubble Plot was analyzed on the online tool of Majorbio Cloud Platform (https://cloud.majorbio.com/page/tools/). Random forest machine learning classification was conducted using the “randomForest” R package with 500 trees, and tenfold cross validation to identify optimal microbial biomarkers.

### *E. coli K 12* and *B. ovatus* culture and growth curves

*B. ovatus (ATCC 8483)* was streaked onto a brain-heart infusion (BHI, OXOID) agar plate in a 37 °C anaerobic work station (10% CO_2_, 10% H_2_ and 80% N_2_) for 48 h. A single colony was used to inoculate 5 mL BHI (supplemented 5% FBS), and seed cultures were suspended 7–8 h at 37 °C to its logarithmic growth period. *Escherichia coli K-12 MG1655* was streaked onto a Luria-Bertani (LB, invitrogen) broth agar plate in a 37 °C aerobic workstation for 48 h. A single colony was seed cultures were suspended 3–4 h at 37 °C to its logarithmic growth period. Bacteria were seeded in 96 multiwell plates in the presence or absence of metformin (40 μM, 1.5 mM and 10 mM, Sigma-Aldrich) and OD_600_ values were measured hourly using a BioTekEon microplate spectrophotometer.

For colonization of bacteria, *B. ovatus* suspended in 200 μL of anaerobic PBS (1.0 × 10^8^ colony-forming units per mouse) were orally administered to mice each day for 10 weeks.

### The quantification of cyanocobalamin in bacteria

The concentration of cyanocobalamin in bacteria was measured by a highly sensitive and selective liquid chromatography coupled with tandem mass spectrometry (LC–MS/MS; AB SCIEX, USA, Triple Quad 6500 + ). In brief, *E. coli* was grown in LB medium and incubated aerobically at 37˚C overnight, and then was maintained and expanded in an M9 medium without cobalamin. The *E. coli* was treated with 3.7 nM cyanocobalamin (Sigma-Aldrich) and metformin (0, 40 μM, 1.5 mM and 10 mM) in the dark for 1 h. *B. ovatus* was grown in minimal media (KH_2_PO_4_ (100 mM), NaCl (15 mM), (NH_4_)_2_SO_4_ (8.5 mM), L-cysteine (4 mM), VK3 (1 μg/mL), hematin (1.9 μM), L-histidine (200 μM), MgCl_2_ (100 μM), FeSO_4_.7H_2_O (1.4 μM), CaCl_2_ (50 μM), Glucose (0.50%)) supplemented where specified with 3.7 nM cyanocobalamin and metformin (0, 40 μM, 1.5 mM and 10 mM) in the dark for 7 h. Next, bacteria were pelleted and washed with 1× PBS (pH 7.4) three times, and 100 μL of MilliQ water containing internal standards (I.S., methotrexate, 100 ng/mL, Sigma-Aldrich) was added to the pellet of bacteria. Then the mixture with 300 μL of cold 20:80 H_2_O: MeOH (0.1% ascorbic acid and beta-mercaptoethanol) was vortexed and sonicated. Next, 400 μL of cold MeOH was added and vortexed again to store at −80 °C for 8 h. Supernatants were collected, dried down under N_2_ flow, resuspended in mobile phase, and kept at 4 °C in an autosampler. An aliquot of 3 μL supernatant was analyzed by LC-MS/MS. The flow rate was set to 0.3 mL/min. Chromatographic separation was performed on a ACQUITY UPLC HSST3 C18 (1.8 μm, 2.1 mm*100 mm) analytical column. The mobile phase included a mixture of 0.1% formic acid in water (A) and acetonitrile (B). The gradient elution was applied and MS detection proceeded in positive mode. The data was collected with a multiple reaction monitor. The MS/MS parameters optimized for the method were showed in Supplementary Table 2. The gradient was set as follows: 10%–30% B at 0–4 min, 30%–10% B at 4–5 min The acquisition data was analyzed using Analyst Software for peak integration, calibration equations and quantification of cyanocobalamin. The protein concentrations were quantitated by 2-D Quant Kit (GE Healthcare) to normalize the amount of bacteria.

### Method validation

(i) The lower limit of quantification (LLOQ) and linearity: The peak area ratios for these compounds and IS were calculated to construct the calibration curves with weighted least squares linear regression (1/x). The LLOQ was the lowest concentration, which could be quantified with an acceptable precision and accuracy. And the peak intensity of it were at least the signal-to-noise ratios (S/N) of about 10. Data was showed at Supplementary Table 3. (ii) Intra- and inter-day precision and accuracy: The high, middle, low concentration levels of quality control samples were prepared to evaluated the intra- and inter-day precision and accuracy, which the ratio of determined concentration to the added concentration must be within 85%–115%, within a day or in three consecutive days. The intra- and inter-day precision and accuracy for all those analytes were exhibited in Supplementary Table 4. (iii) Extraction recovery and matrix effect: Evaluation of a Suitable Extractant was assessed by calculating the extraction recovery and matrix effect. The extraction recovery was calculated using the analyte/IS peak area ratio (A1) detected from extracted biological samples versus with those (A2) spiked post-extraction from these QC sample solutions at the same concentrations. The matrix effects were presented by the ratio of the peak areas of A2 and those of the working solutions containing equivalent amounts of these compounds (A3). Extraction recovery and matrix effect of all compounds were provided in Supplementary Table 5.

### Bacterial RNA extraction and RNA sequencing

Total RNA was extracted from *E. coli* or *B. ovatus* using RNeasy Protect Bacteria Mini Kit (Qiagen) and sequenced on the illumina novaseq 6000 platform, and 150 bp paired-end reads were generated. Three biological replicate cultures of *B. ovatus or E. coli* were grown under metformin (0 mM or 1.5 mM) for 7 h. The sequencing reactions of the twelve samples each produced between 24.2 and 30.9 million clean reads; all samples had ∼93% of bases with a quality score of greater than 30. The RNA-seq data was evaluated using FastQC. The cleaned reads were mapped to the reference genome (GCF_000154125.1_ASM15412v1_genomic.fna and GCF_000005845.2_ASM584v2_genomic.fna) using Bowtie2 (http://bowtie-bio.sourceforge.net/index.shtml). HTSeq 0.6.1p2 (http://www-huber.embl.de/users/anders/HTSeq) was used to get total Read Count associated with a gene. The differentially expressed genes were evaluated by both DESeq2 and edgeR (*P* value was less than or equal to 0.05). Volcano plot was created in R 4.2.0 and the R packages (ggpubr and ggthemes).

### The determination of bacterial ATP level

*E. coli* was grown in LB medium and incubated aerobically at 37 °C for 7 h, then treated with metformin (0, 40 μM, 1.5 mM and 10 mM) in the dark for 1 h. *B. ovatus* was grown in BHI medium (supplemented 5% FBS) supplemented with metformin (0, 40 μM, 1.5 mM and 10 mM) in the dark for 7 h, and then incubated with vehicle or oligomycin A (2 μM) for 1 h. Intracellular ATP level was determined using the ATP Assay Kit (Beyotime). And the protein concentrations of bacteria homogenates were quantitated by BCA assay (Thermo Fisher Scientific).

### Measurement of bacterial membrane potential

*E. coli* was grown in LB medium and incubated aerobically at 37 °C for 7 h. And then it was treated with metformin (0 and 1.5 mM) in the dark for 1 h. *B. ovatus* was grown in BHI medium (supplemented 5% FBS) supplemented with metformin (0, 40 μM, 1.5 mM and 10 mM) in the dark for 7 h. Bacterial membrane potential were detected using BacLight™ Bacterial Membrane Potential Kit (Thermo Fisher Scientific). In brief, *E. coli* or *B. ovatus* were resuspended in 1X PBS and treated with either 10 mM EDTA for 5 min or 10 min. The EDTA-treated bacteria were centrifuged and resuspended in 1X PBS. A 3 mM DiOC_2_(3) stock in DMSO was added to bacteria (final volume of 200 μL in a 96-well opaque microplate) for a final concentration of 30 μM. 15 min after incubation at room temperature, membrane potential was detected by a fluorescent reader under Ex450/Em670 nm.

### Measurement of NADH/NAD^+^ ratio

*E. coli* was grown in LB medium and incubated aerobically at 37 °C for 7 h. And then it was treated with metformin (0 and 1.5 mM) and/or terminal electron acceptors (DMSO (1%); TMAO (4 μM) and NaNO_2_ (18 mM)) in the dark for 1 h. *B. ovatus* was grown in BHI medium (supplemented 5% FBS) supplemented with metformin (0 and 10 mM) in the dark for 7 h. Total intracellular NAD^+^ and NADH levels were measured by using a Amplite™ Fluorimetric NAD^+^/NADH Ratio Assay Kit *Red Fluorescence. Briefly, bacteria were homogenized in lysis buffer and then centrifuged. Next, supernatant in NAD^+^ or NADH extraction buffer was heated to 37 °C for 15 min, and same volume of opposite extraction buffer were added to neutralize the sample. After incubation at room temperature for 1 h, fluorescence increase of standards or samples could be detected by a fluorescent reader under Ex540/Em590 nm.

### Animal gut microbiota DNA extraction and RT-qPCR analysis

Fecal and small intestine contents genomic DNA was extracted using QIAamp PowerFecal Pro DNA kit. The RT-qPCR was conducted to quantify the relative abundance of *B. ovatus*.

### RNA isolation and RT-qPCR analysis

RNA was extracted from bacteria using a hot-phenol method. And RNA was extracted from tissues with Trizol reagent (invitrogen) using a standard chloroform-isoamyl alcohol extraction. 1 μg of total RNA was reverse-transcribed using a transcription kit according to the manufacturer’s instructions. Relative mRNA expression levels were determined by qPCR using the QuantStudio 5 Real-Time PCR system (Thermo Fisher Scientific). The primer sequences are summarized in Supplementary Table 6.

### Animals

Male SPF mice of the C57BL/6J strain (Hunan SJA Laboratory Animal Co., Ltd) were housed in a pathogen-free animal facility under standard laboratory conditions (12 h light and dark cycles, with the temperature kept at 21–24 °C and the humidity at 40–70%) and given free access to food and water. The mice were fed an antibiotics cocktail consisting of vancomycin 50 mg/kg, neomycin 100 mg/kg, metronidazole 100 mg/kg, amphotericin-B 1 mg/kg, and ampicillin 1 mg/mL for 7 days. The gut microbiota-depleted mice (aged 6 weeks) were transplanted with *B. ovatus* or PBS and fed a high-fat diet (HFD, 60% kcal from fat, D12492, Research Diets) for 10 weeks continuously.

Twenty mice were randomly divided into four groups and orally administered the following agents (*n* = 5 mice/group): (1) HFD group: HFD + vehicle; (2) Metformin group: HFD + metformin (200 mg/kg/day, 4 weeks, p.o.); (3) *B. ovatus* group: control vehicle; (4) *B. ovatus* + MET group: metformin (200 mg/kg/day, 4 weeks, p.o.).

Animals were anaesthetized with isoflurane to collect blood samples by retro-orbital sinus puncture. Other specimens (liver, ileum, small intestine contents, colon contents, pancreas and adipose) were collected and stored at −80 °C until analysis.

### Mouse plasma VB12, MMA and Hcy quantification

The level of total plasma VB12, MMA and Hcy were measured using the Mouse VB12 ELISA kit (J&L Biological), Mouse Methylmalonic acid ELISA kit (J&L Biological) and Mouse Hcy ELISA kit (mlbio), respectively.

### The relative quantification of folic acid in mice plasma

Folic acid in plasma were detected by ultra-performance liquid chromatography coupled to a tandem mass spectrometry (LC-MS/MS) method (AB SCIEX, USA, Triple Quad 6500+). The flow rate was set to 0.2 mL/min. Chromatographic separation was performed on a ACQUITY UPLC HSST3 C18 (1.8 μm, 2.1 mm*100 mm) analytical column. The mobile phase included a mixture of 0.1% formic acid in water (A) and acetonitrile (B). The gradient elution was applied and MS detection proceeded in positive mode. The data was collected with a multiple reaction monitor. The MS/MS parameters optimized for the method were showed in Supplementary Table 7. The gradient was set as follows: 5%–5% B 0–0.5 min, 5%–80% B 0.5–5 min, 80%–80% B 5–6 min, 80%–5% B 6–6.1 min, 5%–5% B 6.1–8 min. An aliquot of 50 μL plasma sample was mixed with 150 μL of cold 20:80 H_2_O: MeOH (0.1% ascorbic acid and formic acid) containing internal standard (I.S.), vortexed and then centrifuged at 13,000 *g*, 4 °C for 10 min. An aliquot of 2 μL supernatant was analyzed by LC-MS/MS. The relative quantification was calculated using the analyte/I.S. peak area ratio detected from extracted biological.

### Histological analysis

Liver tissues were fixed in 4% paraformaldehyde and stained by oil red O (ORO). Adipose tissues were fixed in 4% paraformaldehyde and stained by immunostaining with adiponectin antibody (Bioss, Cat# bs-0471R, 1:200). The adipose tissues sections were also stained with hematoxylin and eosin (H&E). For immunofluorescence staining, slides were incubated with insulin (Proteintech, Cat# 15848-1-AP, 1:100) and glucagon (Proteintech, Cat# 67286-1-Ig, 1:200) antibody and counterstained with DAPI to obtain images.

### Western blot analysis

Protein of tissues was separated by SDS-PAGE electrophoresis and transferred to a PVDF membrane. The membrane was incubated with primary antibodies against AHR (Proteintech, Cat# 17840-1-AP, 1:1000), CYP1A1 (Proteintech, Cat# 13241-1-AP, 1:1000), or GAPDH (Cell Signaling Technology, Cat# 2118, 1:20000) overnight at 4 °C.

### Ileal RNA sequencing and analysis

Total RNA was extracted from the mouse ileal tissue using the TRIzol reagent. Samples were subjected to 150 bp paired-end sequencing using the Illumina novaseq 6000 platform. Clean reads were obtained by removing reads containing adapter, reads containing N base and low-quality reads (Q_phred_ <=20) from raw data. Reads were then aligned to the mouse (Mus_musculus.GRCm39.dna.primary assembly.fa) genome using the Hisat2 (version 2.0.5), and featureCounts (version 1.5.0-p3) was used to count the reads numbers mapped to each gene. The average read coverage was 43,675,559 reads/sample with an average of 96% of the reads successfully mapping to the mouse genome. Sequenced reads were normalized to FPKM, and differentially expressed genes between *B. ovatus* + MET and Metformin or *B. ovatus* mouse ileal tissues were determined using DESeq2 R package. Enrichment analysis were performed using clusterProfiler (version 3.4.4). The local version of the Gene Set Enrichment Analysis (GSEA) tool http://www.broadinstitute.org/gsea/index.jsp, GO, KEGG, Reactome, DO and DisGeNET data sets were used for GSEA independently. *P* values of the multiple-comparisons were adjusted with original FDR method of Benjamini and Hochberg, FDR < 0.05. Corrected *P*-value of 0.05 and absolute foldchange of 2 were set as the threshold for significantly differential expression.

### Statistics

GraphPad Prism version 8.0, IBM SPSS Statistics 26 and R (version 3.5.1 and 4.2.0) were used for statistical analysis. Experimental data were shown as the mean ± SEM or mean ± SD. The sample size was estimated on the basis of previous experience, sample availability and previously reported studies. No data were excluded from the data analysis. Unpaired independent Student’s *t* tests (between two groups) and one-way ANOVA with Tukey’s or Dunnett’s tests (among multiple groups) were used to compare differences upon normally distributed and homogeneous variances. Non-normally distributed or heterogeneous data were compared by the Mann–Whitney *U* tests (Wilcoxon rank-sum test, between two groups) or the Kruskal–Wallis test (among multiple groups). A Benjamini-Hochberg adjusted *P*-value (FDR) of 0.05 was used as the cutoff for statistical significance unless stated otherwise. Correlation analysis of gut microbiome and subjects’ clinical characteristics were investigated using *Spearman’s* rank correlation coefficient test. Statistical significance is indicated by asterisks (*): *P < 0.05, **P < 0.01, ***P < 0.001, ****P < 0.0001, ns, non-significant.

### Reporting summary

Further information on research design is available in the Nature Research Reporting Summary linked to this article.

## Supplementary information

Supplemental Material

Reporting Summary

## Data Availability

The raw metagenome data and bacterial RNA sequencing reads are accessible via the NCBI Sequence Read Archive (PRJNA910923 and PRJNA989277, respectively). All other data needed to evaluate the conclusions in the paper are available from the corresponding author upon reasonable request.

## Code availability

No new R analysis scripts or codes were created in this study.
