# Supplementary material for: Bacteroides ovatus accelerates metformin-induced vitamin B12 deficiency in type 2 diabetes patients by accumulating cobalamin
Source: NPJ Biofilms Microbiomes. 2023 Jul 24;9:51. doi: 10.1038/s41522-023-00419-y (PMC10366088; doi:10.1038/s41522-023-00419-y)

Supplemental material for

Bacteroides ovatus accelerates metformin-induced vitamin B12 deficiency in type 2 diabetes patients by accumulating cobalamin

Manyun Chen, Yan Shu, Qing Li, Zhiqiang Kang, Tao Liu, Honghao Zhou, Weihua Huang* and Wei Zhang*.

Correspondence to: csuzhangwei@csu.edu.cn (W.Z.) or endeavor34852@csu.edu.cn (W.H.H.)

This file includes:

Supplementary Fig. 1 to 5 for multiple supplementary figures
Supplementary Table. 1 to 7 for multiple supplementary tables
unmodified gels

1. Supplementary Figures

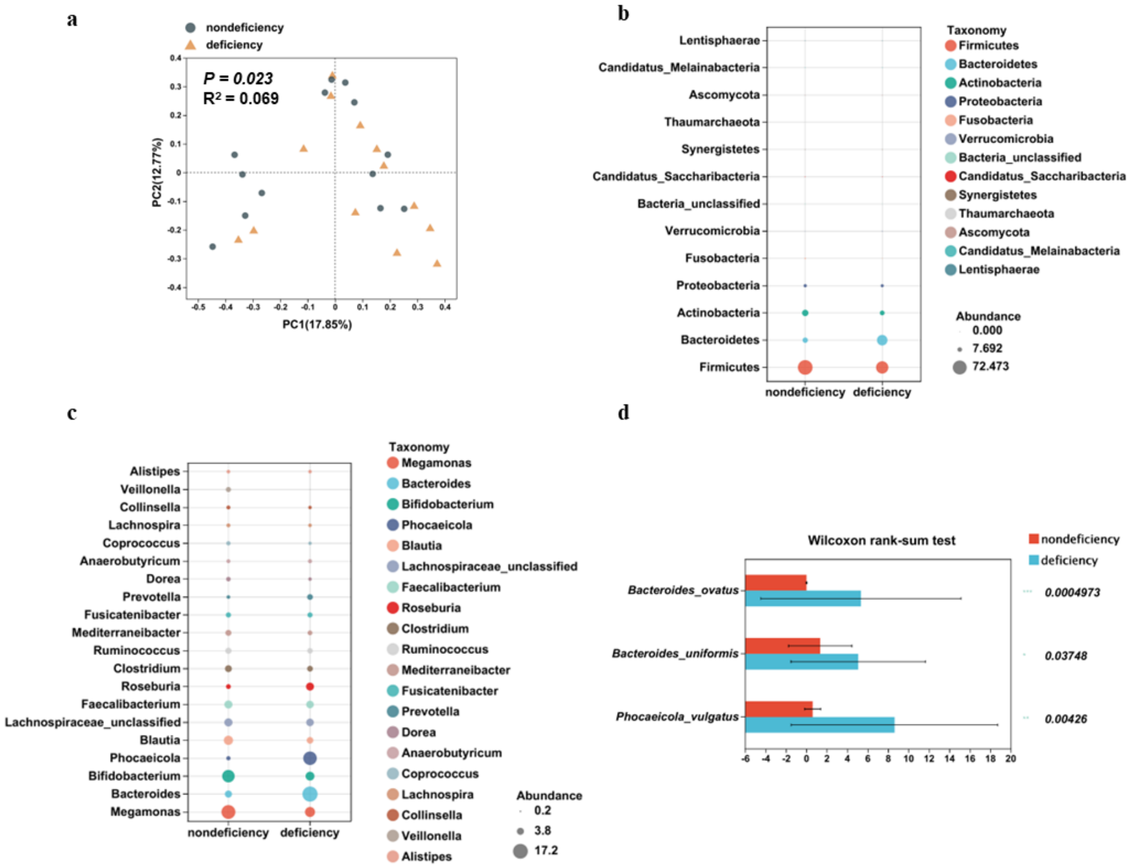

Supplementary Figure. 1 Gut microbiota profiling in patients with VB12 deficiency and non-deficiency after metformin treatment. (**a**) PCoA plot based on the relative abundances at species level according to the *Bray–Curtis* distance; The deficiency group is shown in orange, and the non-deficiency group is shown in green; PERMANOVA test with the *Bray-Curtis* distance was used to assess the significant difference between the two groups, and the result showed significant separation of the deficiency and non-deficiency groups ($P = 0.023$). (**b-c**) Bacterial taxonomic profiling of the gut microbiota from deficiency and non-deficiency groups processed at the phylum and genus levels. (**d**) Abundance of *B. ovatus, B. uniformis* and *P. vulgatus* based on metagenomics data. Wilcoxon rank-sum tests were calculated and then adjusted the *P*-value for a Benjamini-Hochberg FDR.

a

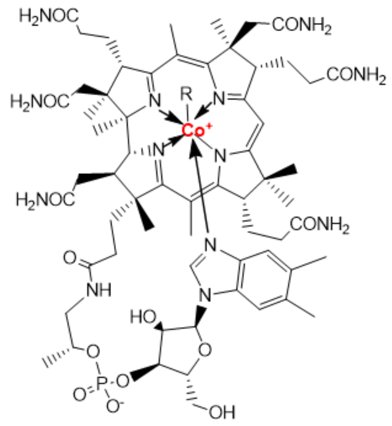

R = 5'-deoxyadenosyl, CH_3, OH, CN

b

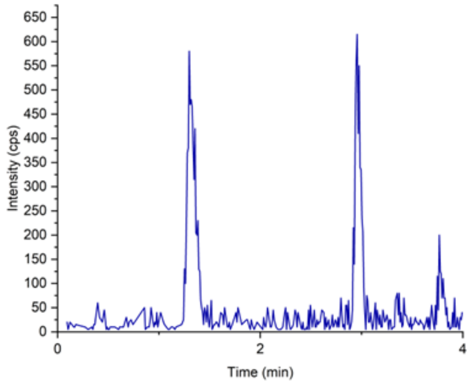

c

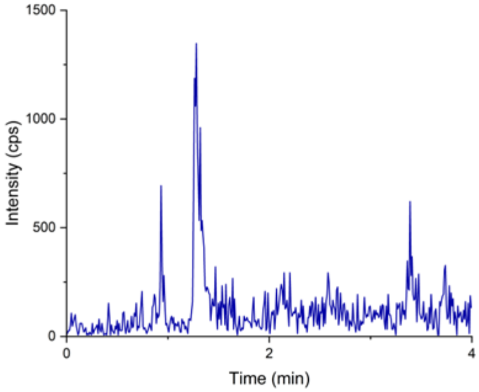

d

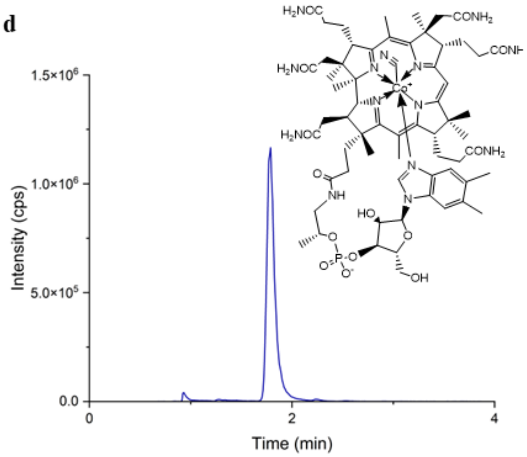

e

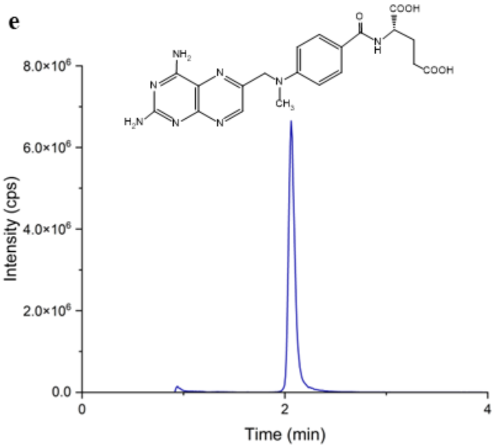

Supplementary Figure. 2 Quantification of intracellular vitamin B12 concentrations of bacteria by LC-MS/MS. (**a**) Chemical structures of vitamin B12. (**b-c**) Typical MRM chromatograms of blank bacterial homogenate. (**d-e**) Typical MRM chromatograms of blank bacterial homogenate spiked with cyanocobalamin or with I.S.

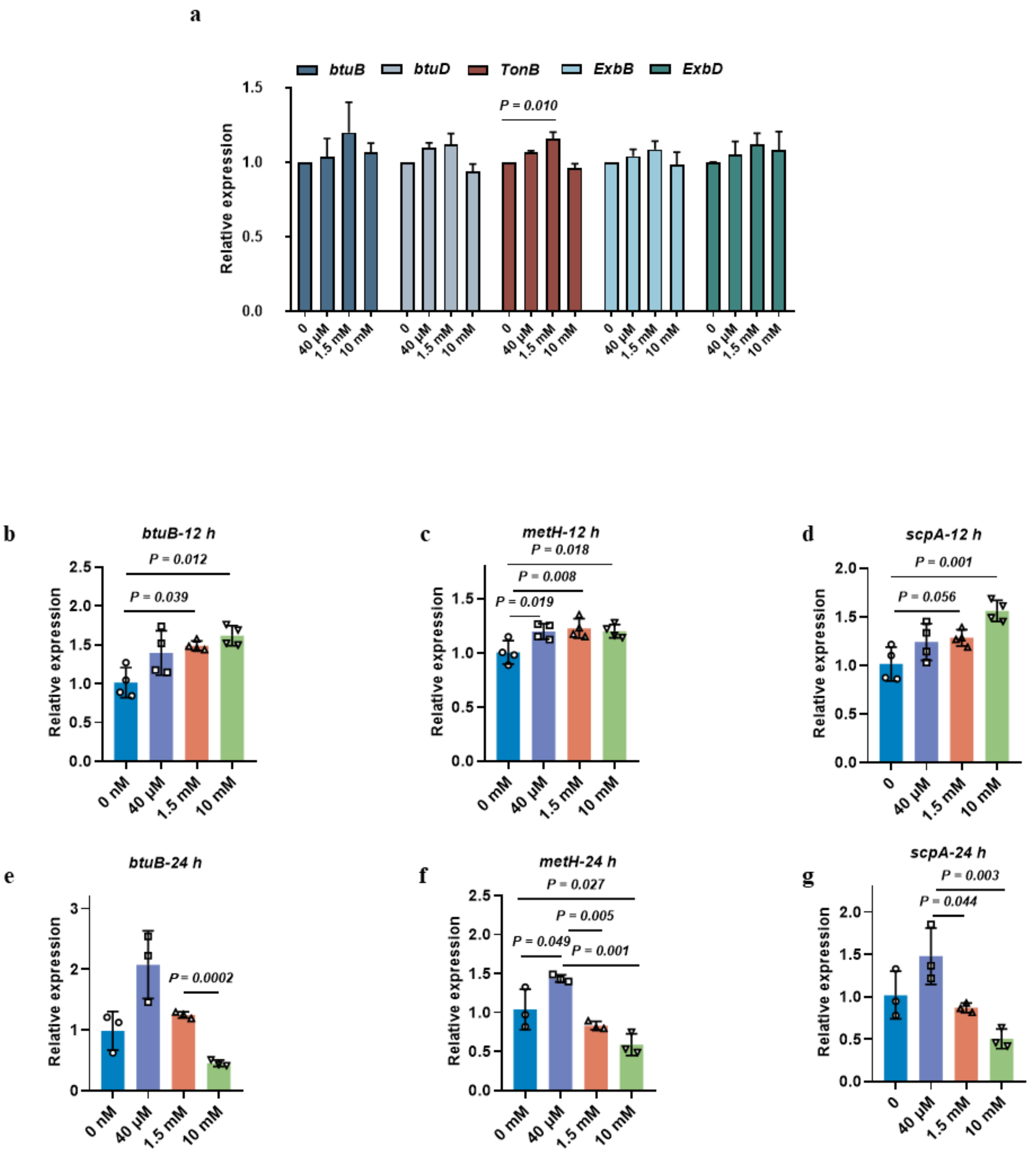

Supplementary Figure. 3 Metformin regulated the expression of VB12 transport genes and VB12-dependent genes of bacteria. (**a**) Relative mRNA abundances of *btuBFCD* and *TonB* complex genes of *E. coli* treated with metformin for 1 hour (n = 3). (**b-d**) Relative mRNA abundances of *btuB*, *metH* and *scpA* of *B. ovatus* treated with metformin for 12 hours (n = 4). (**e-g**) Relative mRNA abundances of *btuB*, *metH* and *scpA* of *B. ovatus* treated with metformin for 24 hours (n = 3). *P* value determined by the one-way ANOVA. All data are presented as mean ± SEM or mean ± SD.

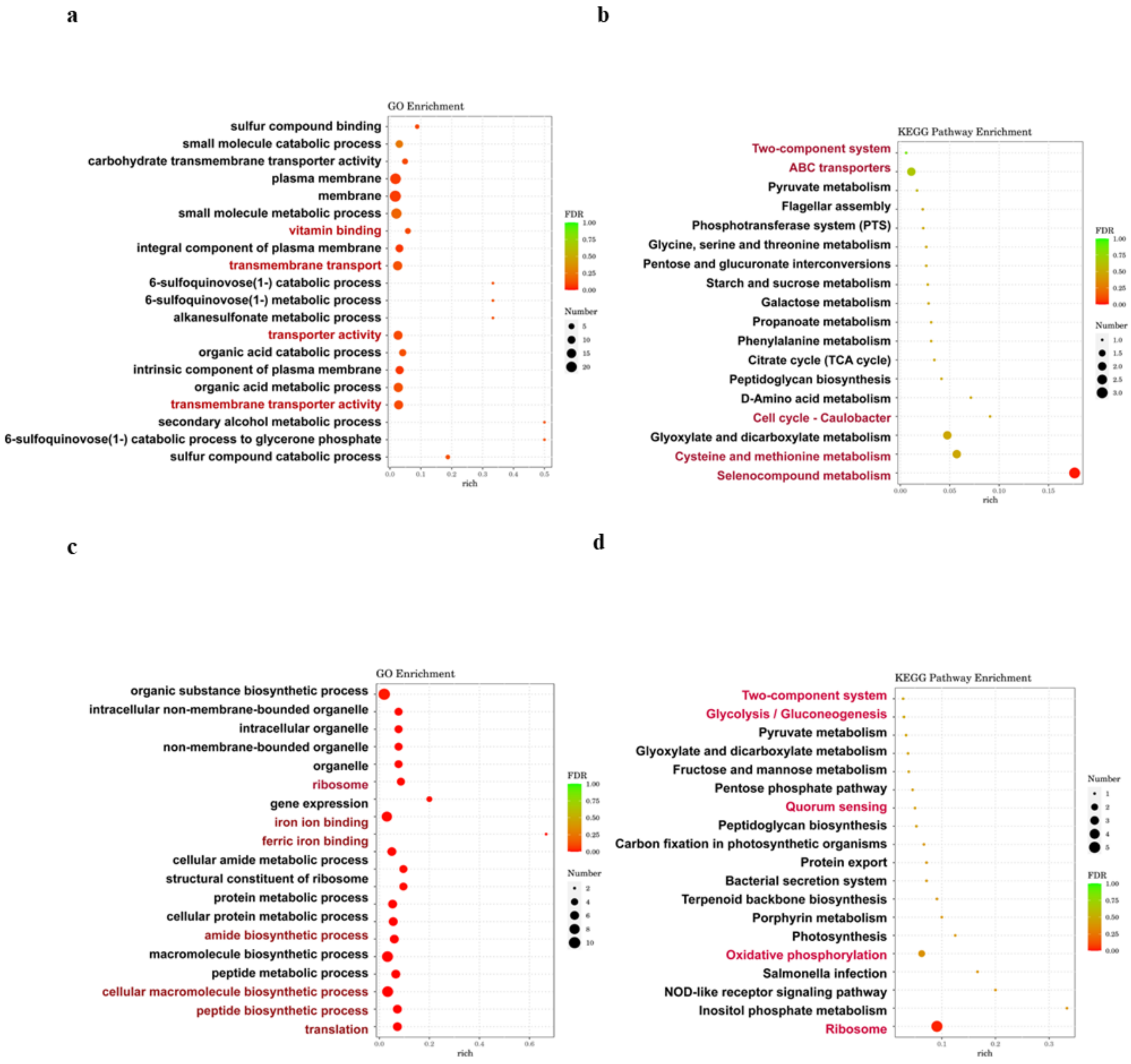

Supplementary Figure. 4 The effects of metformin on *E. coli* or *B. ovatus* by transcriptomics. (**a-b**) Enrichment analysis for up-regulated genes between *E. coli* + 1.5 mM metformin vs *E. coli* + vehicle group was performed in GO and KEGG databases, respectively (n =3). (**c-d**) Enrichment analysis for up-regulated genes between *B. ovatus* + 1.5 mM metformin vs *B. ovatus* + vehicle group was performed in GO and KEGG databases, respectively (n =3).

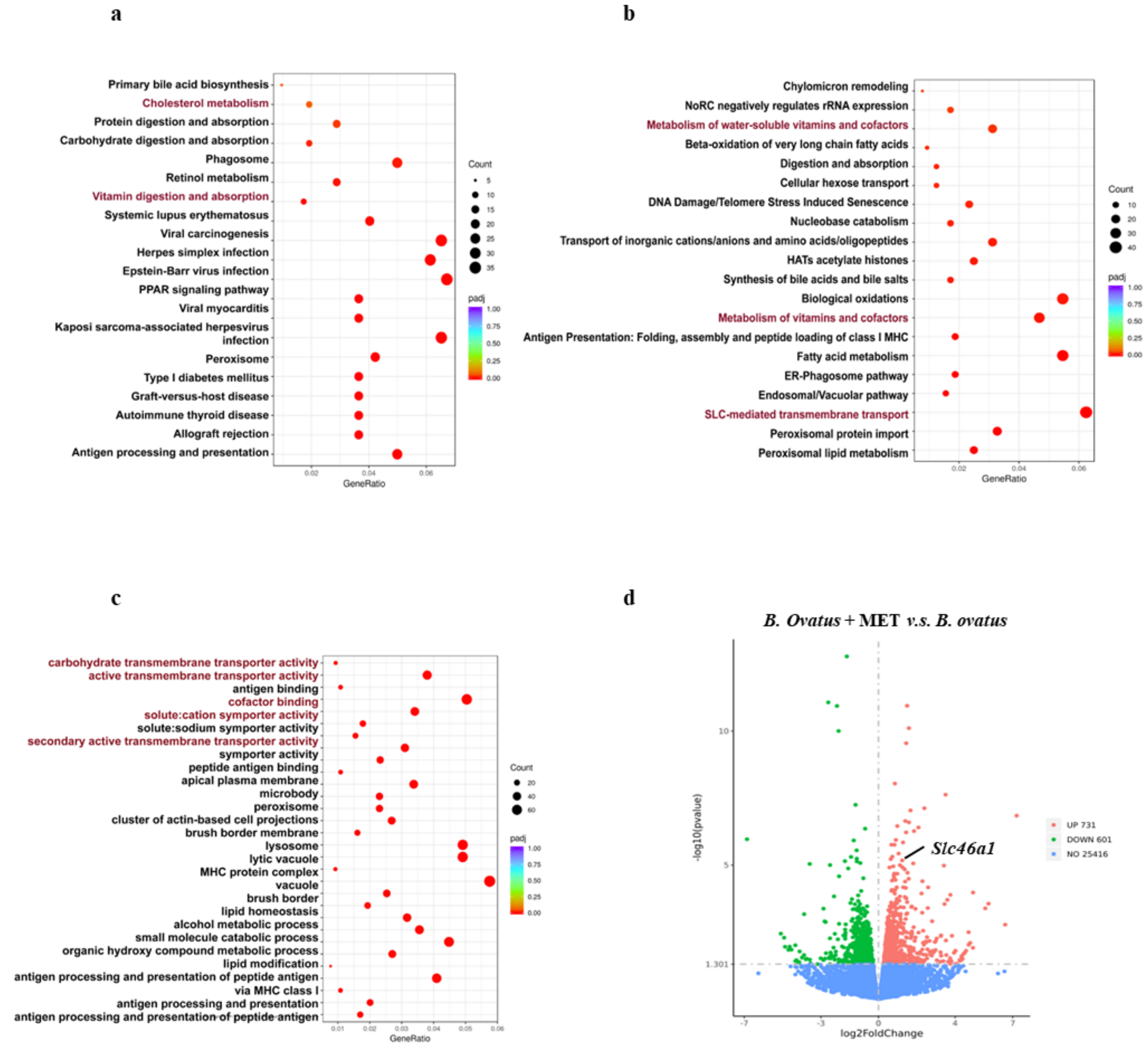

Supplementary Figure. 5 Pathway enrichment analysis of RNA sequencing of mouse ileum were enriched in the target pathway of vitamin digestion and absorption. (**a-c**) Enrichment analyses for up- and down-regulated genes between *B. ovatus* + MET vs metformin mice ileum samples were performed in KEGG, Reactome and GO database, respectively (n =3). (**d**) Volcano plot of significantly upregulated (red), downregulated (green) genes and non-significant (blue) genes in *B. ovatus* + MET and *B. ovatus* group mice (n = 3).

2. Supplementary table 1 the demographics and clinical characteristics of metformin monotherapy patients with naive T2DM (All data presented as mean ± SD)

Clinical characteristics	deficiency (n = 13)	non-deficiency (n = 13)	*P* value
**General Information**			
Age (years)	53.8±8.8	48.7±10.3	*0.190*
Sex (Females/Males), %	38/62	38/62	-
**Anthropometric markers**			
Weight (kg)	69.6±8.9	74.9±8.6	*0.133*
Height (cm)	165.9±8.9	170.7±8.3	*0.170*
BMI (Body mass index)	25.2±2.1	25.7±2.4	*0.575*
Waist circumference (cm)	90.7±7.4	92.1±7.7	*0.640*
Hip circumference (cm)	95.8±6.7	95.9±8.2	*0.967*
SBP (mmHg)	127.1±11.6	124.6±14.6	*0.628*
DBP (mmHg)	74.7±8.0	78.1±13.0	*0.430*
**Laboratory tests**			
WBC ($\times 10^9$/L)	6.59±1.20	6.91±1.91	*0.618*
NC ($x10^9$/L)	4.03±1.16	4.48±1.54	*0.422*
Hb (g/L)	140.31±11.81	140.17±16.35	*0.980*
PLT ($\times 10^9$/L)	256.69±70.76	257.15±53.25	*0.985*
Total Bilirubin (μmol/L)	16.92±7.53	15.70±5.82	*0.649*
Direct Bilirubin (μmol/L)	4.52±1.38	5.05±3.24	*0.587*
ALT (U/L)	25.69±20.65	27.85±12.85	*0.752*
AST (U/L)	21.69±6.49	26.23±7.35	*0.108*
TG (mmol/L)	1.22±0.86	2.06±1.18	*0.047*
TC (mmol/L)	4.01±1.18	4.47±2.93	*0.607*
HDL (mmol/L)	1.28±0.26	1.23±0.35	*0.699*
LDL (mmol/L)	2.44±0.69	2.02±0.89	*0.183*
BUN (mmol/L)	4.88±0.89	4.50±1.03	*0.334*
Cr (μmol/L)	52.35±13.57	51.15±15.45	*0.838*
UA (μmol/L)	290.66±70.58	331.26±74.51	*0.176*
HbA1c (mmol/mol)	54.77±11.98	43.59±9.03	*0.013*
HbA1c (%)	7.16±1.10	6.14±0.83	*0.013*
FPG (mmol/L)	8.21±2.37	7.30±1.97	*0.296*
PPG (mmol/L)	12.58±4.56	8.49±3.32	*0.015*
FINS (μIU/ml)	8.92±5.75	15.95±20.44	*0.244*
**VB12 status**			
VB12 (pg/mL)	135.30±41.95	386.03±189.43	*<0.0001*
Hcy (μmol/L)	12.78±4.13	9.93±4.17	*0.048*
Folate (ng/mL）	9.67±2.26	12.66±5.85	*0.281*

Supplementary table 2 The optimized mass spectrometric parameters of all the compounds

Analyte	Precursor ion (*m/z*)	Product ion (*m/z*)	Dwell Time (ms)	Declustering potential (V)	Entrance potential (V)	Collision energy (eV)	Collision cell exit potential (V)
VB12	678.600	147.100	200	150	10	40	7
I.S.	455.20	308.20	150	115	10	28	15

Supplementary table 3 The linear range, calibration curves with linearity (R^2) and lower limit of quantification (LLOQ)

Compounds	Linear range (ng/ml)	R^2	Calibration curves	LLOQ (ng/ml)
Vitamin B12	0.3975-127.2	0.997	y=0.000528x+0.000142	0.3975

Supplementary table 4 Precision and accuracy for all compounds (All data presented as mean)

Compound	Conc. Added (ng/mL)	interday (n = 6)		intraday (n=18)	
		Accuracy (%)	precision (%)	Accuracy (%)	precision (%)
VB12	0.3975	90.67	3.00	-	-
	0.795	91.67	9.45	99.03	8.68
	6.36	100.98	6.39	105.61	5.41
	101.76	102.35	6.45	106.73	5.01

Supplementary table 5 Extraction recovery and matrix effect for all compounds (All data presented as mean)

Compounds	Conc. Added (ng/mL)	Recovery rate (%) (n= 6)	RSD (%)	Matrix effect (%) (n = 6)	RSD (%)
VB12	0.795	88.02	13.78	127.81	13.08
	6.36	92.77	5.82	106.08	6.83
	101.76	94.23	3.42	103.05	3.42

Supplementary table 6 List of primers

Origins	Targeted genes	Sequences (5’→3’)
**Bacteria**	*B. ovatus*	F: CAGAACGTGTCACCTCTCCT<br>R: CCGTGACAGTTGCAGCATAA
	Total bacteria	F: GTGSTGCAYGGYTGTCGTCA<br>R: ACGTCRTCCMCACCTTCCTC
	ECTonB	F: TGTTGTGGCGGGTCTGCTCTATAC<br>R: CAGGCGTAACCATCGTGACAGAAATC
	ECmetH	F: AACTGGCATTGAAGAGCACAACAAC<br>R: TTGCGAATAGCGTAGTAGAGGAACAC
	ECscpA	F: CCATCAGTATCAGCGGTTACCACAT<br>R: GAAGAAGAACGACAGGCGAGGAG
	ECfdnH	F: TGGAAACGCAGGACATTATCAA<br>R: TTACAGCCGATACAGGTGGAA
	ECmdoG	F: CGGACTATCAGCAGATCCAGTTT<br>R: CGGGCTGTATTTGATTCGTTTGA
	BObtuB	F: ACAAGAACATCATCGGGCAGTATCA<br>R: GTCGGTCGTCAACATCGGAAGT
	BOmetH	F: CGCAAGTTCCTTCGTCTCATCAATG<br>R: GCATCCAGCAATCCGTCATCCA
	BOscpA	F: GTCGGTGTGAACAAGTATCGTCTG<br>R: ATTCGGTAATTGCTGCCAATGCTT
	BOatpA	F: TCTGATTCTTCGCCGTCCAT<br>R: GCCACTTCCTCCTGACTGATA
	BOatpB	F: CGTTGATTCTGTTCTGCCTCAT<br>R: CCGTGTTCTCCTGTTCCTCTT
	BOatpC	F: CATTTGAGTATAGTATCGCCCGAAA<br>R: GTGTGCTCTTCTCCGTCCAT
	BOatpD	F: TCACGCAAGCAGGTTCAGA<br>R: AAGTCATCGGCAGGCACAT
	BOatpE	F: CAGCAGCAGTAGGAGTTAG<br>R: TAGAGGAAGAACACCAACAG
	BOatpF	F: TGCGGTGAAACAACAAATCCA<br>R: CATCTGTGCTTCCTTGTCCTC
	BOrpoD	F: AGCCTGTTGGATGTGTTGGT<br>R: CCTCGCAAGAGACTCATTAACC

Supplementary table 6 List of primers (*Continued*)

Origins	Targeted genes	Sequences (5'→3')
**Mouse**	*Ahr*	F: AGCCGGTGCAGAAAACAGTAA<br>R: AGGCGGTCTAACTCTGTGTTC
	Cyp1a1	F: GACCCTTACAAGTATTTGGTCGT<br>R: GGTATCCAGAGCCAGTAACCT
	Cubn	F: CACTTTAGGTTGTGGTGGAACA<br>R: TTGCTGTCAAAGCTAATCTCCC
	Tcn2	F: TACCTTGGATGGATCGGCTTT<br>R: GGTAATGGATCTTGAGGCTGTGA
	Mmachc	F: GAGCCAGACTGGTTTCCTATCT<br>R: GCCACACTGTTCGAGAGCATA
	Lmbrd1	F: ATGCAGGCTATTTTGGCCTTT<br>R: TGTACGGTGACATTGGCGTC
	Abcc1	F: TGATGGCTCCGATCCACTCT<br>R: TCCACAGAAAGAATCCTAAGGCA
	Slc19a1	F: CTCTTTCTAAAGCGCCCTAAGC<br>R: AGGACATGCACATAGTAGGTGA
	Slc46a1	F: CCGTGGAGCCCCTAGTCTT<br>R: GGGTGCCATTGTAGCCGAG
	GAPDH	F: GGTTGTCTCCTGCGACTTCA<br>R: TGGTCCAGGGTTTCTTACTCC
	β-actin	F: CTAAGGCCAACCGTGAAAAG<br>R: ACCAGAGGCATACAGGGACA

Supplementary table 7 The optimized mass spectrometric parameters of all the compounds

Analyte	Precursor ion (*m/z*)	Product ion (*m/z*)	Dwell Time (ms)	Declustering potential (V)	Entrance potential (V)	Collision energy (eV)	Collision cell exit potential (V)
Folate	442.30	295.30	150	60	10	28	16
I.S.	455.20	308.20	150	115	10	28	15

Data S1. Original Images of Representative Western. Related to Figures 5.

Fig 5b

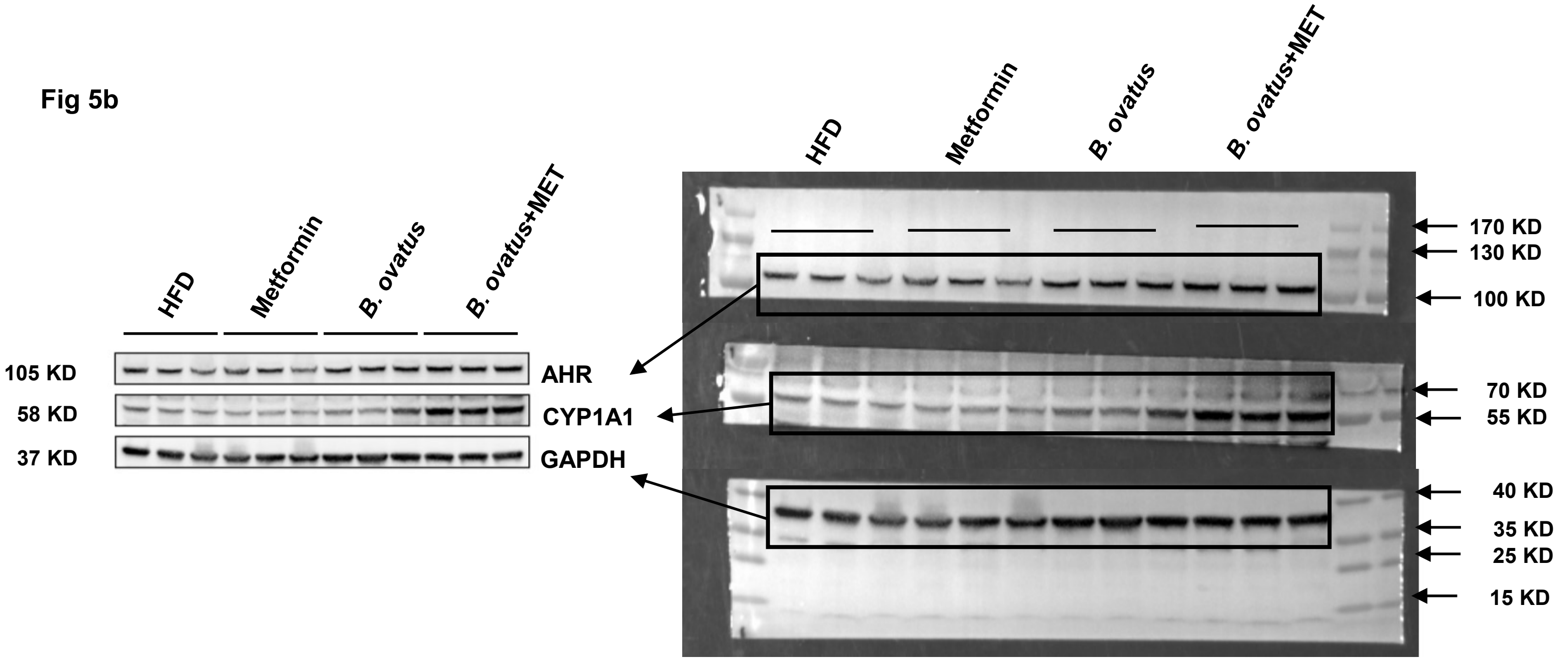

Supplement: Supplementary file 1 — Supplemental Material [file 41522_2023_419_MOESM1_ESM.pdf]
