# Supplementary material for: Bacteroides ovatus accelerates metformin-induced vitamin B12 deficiency in type 2 diabetes patients by accumulating cobalamin
Source: NPJ Biofilms Microbiomes. 2023 Jul 24;9:51. doi: 10.1038/s41522-023-00419-y (PMC10366088; doi:10.1038/s41522-023-00419-y)
Supplement: Supplementary file 2 — Reporting Summary [file 41522_2023_419_MOESM2_ESM.pdf]

nature portfolio

Corresponding author(s): Weihua Huang and Wei Zhang

Last updated by author(s): Jul 1, 2023

Reporting Summary

Nature Portfolio wishes to improve the reproducibility of the work that we publish. This form provides structure for consistency and transparency in reporting. For further information on Nature Portfolio policies, see our Editorial Policies and the Editorial Policy Checklist.

Statistics

For all statistical analyses, confirm that the following items are present in the figure legend, table legend, main text, or Methods section.

n/a	Confirmed	
☐	☒	The exact sample size (*n*) for each experimental group/condition, given as a discrete number and unit of measurement
☐	☒	A statement on whether measurements were taken from distinct samples or whether the same sample was measured repeatedly
☐	☒	The statistical test(s) used AND whether they are one- or two-sided<br>*Only common tests should be described solely by name; describe more complex techniques in the Methods section.*
☒	☐	A description of all covariates tested
☐	☒	A description of any assumptions or corrections, such as tests of normality and adjustment for multiple comparisons
☐	☒	A full description of the statistical parameters including central tendency (e.g. means) or other basic estimates (e.g. regression coefficient) AND variation (e.g. standard deviation) or associated estimates of uncertainty (e.g. confidence intervals)
☐	☒	For null hypothesis testing, the test statistic (e.g. *F*, *t*, *r*) with confidence intervals, effect sizes, degrees of freedom and *P* value noted<br>*Give P values as exact values whenever suitable.*
☒	☐	For Bayesian analysis, information on the choice of priors and Markov chain Monte Carlo settings
☒	☐	For hierarchical and complex designs, identification of the appropriate level for tests and full reporting of outcomes
☒	☐	Estimates of effect sizes (e.g. Cohen's *d*, Pearson's *r*), indicating how they were calculated

Our web collection on statistics for biologists contains articles on many of the points above.

Software and code

Policy information about availability of computer code

Data collection

Metagenomics sequencing was performed on the illumina novaseq platform (Illumine). RNA sequencing was performed on the illumina novaseq 6000 platform (Illumine).
Concentration of the cyanocobalamin was detected by an UPLC-MS/MS (AB SCIEX, Triple Quad 6500+).
OD600 values of bacteria were measured by Microplate Spectrophotometer (BioTek, BIOTEK-EON).
H&E, immunohistochemistry and immunofluorescence images were captures using Pannoramic MIDI (3DHISTECH).
qRT-PCR results were acquired using the QuantStudio 5 Real-Time PCR system (Thermo Fisher Scientific).
The absorbance values for ELISA or triglyceride measurements were acquired by Microplate Spectrophotometer (Thermo Fisher Scientific, Varioskan LUX).
Western blot images were captures using Molecular Imager ChemiDoc™ XRS+ imaging System (BIO-RAD, Universal Hood II).
Luciferase assays were performed using the dual luciferase assay system (Promega). Firefly and Renilla luciferase activities were measured by Veritas microplate illuminometer (Turner Biosystems).

Data analysis

Software packages used in metagenomics and RNA sequencing analysis were detailed in Methods and Supplementary material, including Bowtie2, Trim Galore, metaSPAdes, QUAST, MetaGeneMark, CD-HIT, samtools-depth, MetaPhlAn 4.0.6, KOBAS 3.0 and R (Version 3.5.1; 4.1.2).
GraphPad Prism version 8.0 (GraphPad Software) and IBM SPSS Statistics 26 (SPSS Inc.) were used for statistical analysis.
Analyst Software 1.6.3 (AB SCIEX) was used for UPLC-MS/MS data analysis.
Gen5 CHS 2.01 Software (BioTek) was used for growth curves monitoring of bacteria.
CaseViewer 2.4 Software (3DHISTECH) and ImageJ (National Institutes of Health) were used for H&E, immunohistochemistry and immunofluorescence image processing.

Skanlt RE 6.1 Software (Thermo Fisher Scientific) was used for ELISA analysis and hepatic triglyceride measurement.
Image LabTM Software (BIO-RAD) and ImageJ (National Institutes of Health) were used in western blot images processing.

For manuscripts utilizing custom algorithms or software that are central to the research but not yet described in published literature, software must be made available to editors and reviewers. We strongly encourage code deposition in a community repository (e.g. GitHub). See the Nature Portfolio guidelines for submitting code & software for further information.

Data

Policy information about availability of data

All manuscripts must include a data availability statement. This statement should provide the following information, where applicable:

- Accession codes, unique identifiers, or web links for publicly available datasets
- A description of any restrictions on data availability
- For clinical datasets or third party data, please ensure that the statement adheres to our policy

The raw metagenome data and bacterial RNA sequencing reads are accessible via the NCBI Sequence Read Archive (PRJNA910923 and PRJNA989277, respectively).

Research involving human participants, their data, or biological material

Policy information about studies with human participants or human data. See also policy information about sex, gender (identity/presentation), and sexual orientation and race, ethnicity and racism.

Reporting on sex and gender	In this study, we use the term sex. And no sex-based analyses have been performed, due to no reports about the association between metformin-induced VB12 deficiency and sex.
Reporting on race, ethnicity, or other socially relevant groupings	No race, ethnicity, or other socially relevant groupings-based analyses have been performed, due to all subjects as Han Chinese.
Population characteristics	Chinese patients with newly diagnosed T2DM were recruited for the study (aged 18-65 years; 18.5 kg/m2 < BMI < 35.0 kg/m2) and has never been treated with anti-diabetic drugs before this study. The population characteristics are described in detail in the Supplementary table 1.
Recruitment	In this study, we conducted a clinical prospective cohort study, consisting of 26 newly diagnosed Type 2 diabetes mellitus patients treated with metformin for 3 to 6 months. The protocol outlines the inclusion exclusion criteria. Patients were excluded if they did not meet the criteria. This helps to minimize bias.
Ethics oversight	The study protocol was approved by the Medical Ethics Committee of Xiangya Hospital, Central South University (ID: 2019040116), and conducted in accordance with the principles of the Declaration of Helsinki. All participants provided written informed consent.

Note that full information on the approval of the study protocol must also be provided in the manuscript.

Field-specific reporting

Please select the one below that is the best fit for your research. If you are not sure, read the appropriate sections before making your selection.

☒ Life sciences ☐ Behavioural & social sciences ☐ Ecological, evolutionary & environmental sciences

For a reference copy of the document with all sections, see nature.com/documents/nr-reporting-summary-flat.pdf

Life sciences study design

All studies must disclose on these points even when the disclosure is negative.

Sample size	Sample sizes were determined based on preliminary experiments and commonly used sample sizes in comparable publications within the field.<br>With in vitro data, sample size used for analysis was 3 to 9.<br>For in vivo data generated using mice, sample size used for analysis was 5 mice per group.<br>Sample size were chosen based on numbers used in similar studies. No power calculations were performed in advance.
Data exclusions	No Data were excluded.
Replication	All experiments were conducted with biological replicates of ≥ 3. All experimental findings were reliably reproduced, and the number of replications were specified in figure legends.
Randomization	The in vitro data were observational findings from different bacteria; thus, randomization was not applicable.<br>Mice were randomly allocated to the study groups and separated in different cages, which had similar baseline characteristics.
Blinding	This was an exploratory study, and as such, there was no treatment group requring blinding.

Reporting for specific materials, systems and methods

We require information from authors about some types of materials, experimental systems and methods used in many studies. Here, indicate whether each material, system or method listed is relevant to your study. If you are not sure if a list item applies to your research, read the appropriate section before selecting a response.

Materials & experimental systems

n/a	Involved in the study
☐	☒ Antibodies
☒	☐ Eukaryotic cell lines
☒	☐ Palaeontology and archaeology
☐	☒ Animals and other organisms
☐	☒ Clinical data
☒	☐ Dual use research of concern
☒	☐ Plants

Methods

n/a	Involved in the study
☒	☐ ChIP-seq
☒	☐ Flow cytometry
☒	☐ MRI-based neuroimaging

Antibodies

Antibodies used	adiponectin antibody (Bioss, Cat# bs-0471R)<br>insulin (Proteintech, Cat# 15848-1-AP)<br>glucagon (Proteintech, Cat# 67286-1-Ig)<br>AHR (Proteintech, Cat# 17840-1-AP)<br>CYP7B1 (Proteintech, Cat# 13241-1-AP)<br>GAPDH (Cell Signaling Technology, Cat# 2118)
Validation	Antibodies were validated by the manufacture, the positive controls in current study, and by correlating Protein Ladder marker (PageRuler Prestained Protein Ladder, cat# 26617, Thermo Scientific).

Animals and other research organisms

Policy information about studies involving animals; ARRIVE guidelines recommended for reporting animal research, and Sex and Gender in Research

Laboratory animals	Male C57BL/6J mice (aged 6 weeks) were purchased from Hunan SJA Laboratory Animal Co., Ltd. (China). Mice were housed in specific pathogen-free conditions (SPF) under alternating 12 hr light and dark cycles and given free access to food and water.
Wild animals	This study did not involve wild animal.
Reporting on sex	Male mice were used in this study, which based on sex used in similar studies.
Field-collected samples	This study did not involve samples collected from the field.
Ethics oversight	The experimental procedures were performed in compliance with Laboratory Animal Center of Central South University guidelines and approved by the Institutional Animal Care and Use Committee (No. CSU-2022-0403).

Note that full information on the approval of the study protocol must also be provided in the manuscript.

Clinical data

Policy information about clinical studies

All manuscripts should comply with the ICMJE guidelines for publication of clinical research and a completed CONSORT checklist must be included with all submissions.

Clinical trial registration	ChiCTR1900022997 (Chinese Clinical Trial Registry)
Study protocol	Participants were administered with metformin hydrochloride treatment (1,000 mg BID for 1-2 weeks, then 2,000 mg per day, Sino-American Shanghai Squibb Pharmaceuticals Ltd.). Such treatment lasted 12-24 weeks, participants were assigned to the deficiency group (serum total VB12 < 200 pg/mL) or to the non-deficiency group (serum total VB12 > 200 pg/mL).
Data collection	Anthropometric measurements, biological samples (plasma and feces samples), and metabolic testing were carried out at the end-of-intervention term. Venous blood and feces samples were prospectively collected at different time points (baseline and at the end-of-intervention term).
Outcomes	The primary endpoint was completion of 3 to 6 months of metformin treatment. The secondary endpoints included Serious Adverse Events (SAEs) , dropout, lost to follow-up.
